# Biokinetics and effects of barium sulfate nanoparticles

**DOI:** 10.1186/s12989-014-0055-3

**Published:** 2014-10-21

**Authors:** Nagarjun Konduru, Jana Keller, Lan Ma-Hock, Sibylle Gröters, Robert Landsiedel, Thomas C Donaghey, Joseph D Brain, Wendel Wohlleben, Ramon M Molina

**Affiliations:** Department of Environmental Health, Molecular and Integrative Physiological Sciences Program, Harvard School of Public Health, 665 Huntington Avenue, Boston, MA 02115 USA; Experimental Toxicology and Ecology, BASF SE, GV/TB - Z470, Carl-Bosch-Straße 38, Ludwigshafen, 67056 Germany

**Keywords:** Lung absorption, Bioavailability, Particokinetics, Particle dissolution, Translocation, Inhalation

## Abstract

**Background:**

Nanoparticulate barium sulfate has potential novel applications and wide use in the polymer and paint industries. A short-term inhalation study on barium sulfate nanoparticles (BaSO_4_ NPs) was previously published [*Part Fibre Toxicol* 11:16, 2014]. We performed comprehensive biokinetic studies of ^131^BaSO_4_ NPs administered via different routes and of acute and subchronic pulmonary responses to instilled or inhaled BaSO_4_ in rats.

**Methods:**

We compared the tissue distribution of ^131^Ba over 28 days after intratracheal (IT) instillation, and over 7 days after gavage and intravenous (IV) injection of ^131^BaSO_4_. Rats were exposed to 50 mg/m^3^ BaSO_4_ aerosol for 4 or 13 weeks (6 h/day, 5 consecutive days/week), and then gross and histopathologic, blood and bronchoalveolar lavage (BAL) fluid analyses were performed. BAL fluid from instilled rats was also analyzed.

**Results:**

Inhaled BaSO_4_ NPs showed no toxicity after 4-week exposure, but a slight neutrophil increase in BAL after 13-week exposure was observed. Lung burden of inhaled BaSO_4_ NPs after 4-week exposure (0.84 ± 0.18 mg/lung) decreased by 95% over 34 days. Instilled BaSO_4_ NPs caused dose-dependent inflammatory responses in the lungs. Instilled BaSO_4_ NPs (0.28 mg/lung) was cleared with a half-life of ≈ 9.6 days. Translocated ^131^Ba from the lungs was predominantly found in the bone (29%). Only 0.15% of gavaged dose was detected in all organs at 7 days. IV-injected ^131^BaSO_4_ NPs were predominantly localized in the liver, spleen, lungs and bone at 2 hours, but redistributed from the liver to bone over time. Fecal excretion was the dominant elimination pathway for all three routes of exposure.

**Conclusions:**

Pulmonary exposure to instilled BaSO_4_ NPs caused dose-dependent lung injury and inflammation. Four-week and 13-week inhalation exposures to a high concentration (50 mg/m^3^) of BaSO_4_ NPs elicited minimal pulmonary response and no systemic effects. Instilled and inhaled BaSO_4_ NPs were cleared quickly yet resulted in higher tissue retention than when ingested. Particle dissolution is a likely mechanism. Injected BaSO_4_ NPs localized in the reticuloendothelial organs and redistributed to the bone over time. BaSO_4_ NP exhibited lower toxicity and biopersistence in the lungs compared to other poorly soluble NPs such as CeO_2_ and TiO_2_.

**Electronic supplementary material:**

The online version of this article (doi:10.1186/s12989-014-0055-3) contains supplementary material, which is available to authorized users.

## Background

Barium sulfate nanoparticles (BaSO_4_ NPs) are used as fillers in coatings (e.g. in motor vehicles) due to their mechanical, optical and chemical properties. Recently, BaSO_4_ NPs have also been used in orthopedic medicine, diagnostic imaging and other applications [1-5]. It has been reported that pellethane, a polyurethane elastomer, when incorporated with BaSO_4_ NPs exhibited antimicrobial properties *in vitro* [6]. Exposure to aerosolized BaSO_4_ NPs may occur during their production, shipping, handling, incorporation into final products, and the use and disposal of those products. Chronic exposure to high levels of micron-scale BaSO_4_ sulfate may induce pneumoconiosis (baritosis) in miners [7-9].

Barium sulfate is considered a member of the poorly soluble particles (PSP) or poorly soluble low toxicity (PSLT) particle groups, as are cerium dioxide (CeO_2_) and titanium dioxide (TiO_2_) [10-12]. These biodurable nanomaterials are usually poorly absorbed after oral and inhalation exposure [13-15]. Particokinetics of nanoparticles are influenced by particle size and route of exposure [16]. Poorly soluble particles may also differ in clearance and biological effects compared to soluble particles [12,17-19]. It is not established whether the biokinetics of inhaled BaSO_4_ NPs are similar to other PSLT NPs. Therefore, it is of interest whether the biokinetics of inhaled BaSO_4_ NPs are different from other PSLT NPs. Previous studies have described the lung clearance of intratracheally instilled micron-sized radioactive BaSO_4_ and showed that the particle size influences lung clearance of Ba [20,21]. A subchronic inhalation study in rats showed a neutrophil increase in bronchoalveolar lavage (BAL) with micron-scale TiO_2_ but not with BaSO_4_ at comparable overload lung burdens (~10 mg Ba) [12,17]. The difference was attributed to the lower surface area of BaSO_4_ than TiO_2_. Toxicity of nanoparticles is influenced by particle physicochemical properties [16,22-24]. The biological responses to small particles differ from bigger particles of the same composition [25,26]. Furthermore, a short-term inhalation study on BaSO_4_ NPs has been reported recently [27,28]. Rats were exposed (nose-only) to 50 mg/m^3^ BaSO_4_ (NM-220) for 6 hours/day for 5 days. It was found that the lung burden of BaSO_4_ at the end of exposure was 1.1 mg/lung which decreased to 0.24 mg/lung within 21 days. This short-term exposure to BaSO_4_ did not elicit significant pulmonary or systemic responses consistent with previous reports in various *in vitro* and *in vivo* test systems [26]. The mechanisms underlying the lower toxicity and rapid lung clearance of BaSO_4_ NPs are not fully understood. For example, more research is needed to quantify the components of clearance attributable to intact particles versus particle dissolution and clearance of barium ions. Thus, there is continuing interest in the biokinetics and effects of BaSO_4_ NPs, especially after pulmonary exposure. A two-year inhalation study of BaSO_4_ and CeO_2_ has been initiated in collaboration between the German Federal Ministry for the Environment German government and BASF (Ludwigshafen, Germany). The project is within the Organization for Economic Cooperation and Development (OECD) sponsorship program and the European Union Project NANoREG (a European approach to the regulatory testing of manufactured nanomaterials).

The data presented here were used in designing this long-term inhalation study. Our objective was to characterize the pulmonary and systemic effects of inhaled BaSO_4_ NPs after short-term and subchronic exposure. In addition, we report here a comprehensive study on the biokinetics of ^131^Ba after intratracheal instillation (IT), intravenous injection (IV) and gavage administration of radiolabeled ^131^BaSO_4_ NPs. These studies are important in assessment of risks from exposure to BaSO_4_ NPs.

## Results

### Physicochemical characterization of NM-220 and the reproduced batch of BaSO_4_ nanoparticles

**Figure 1:**
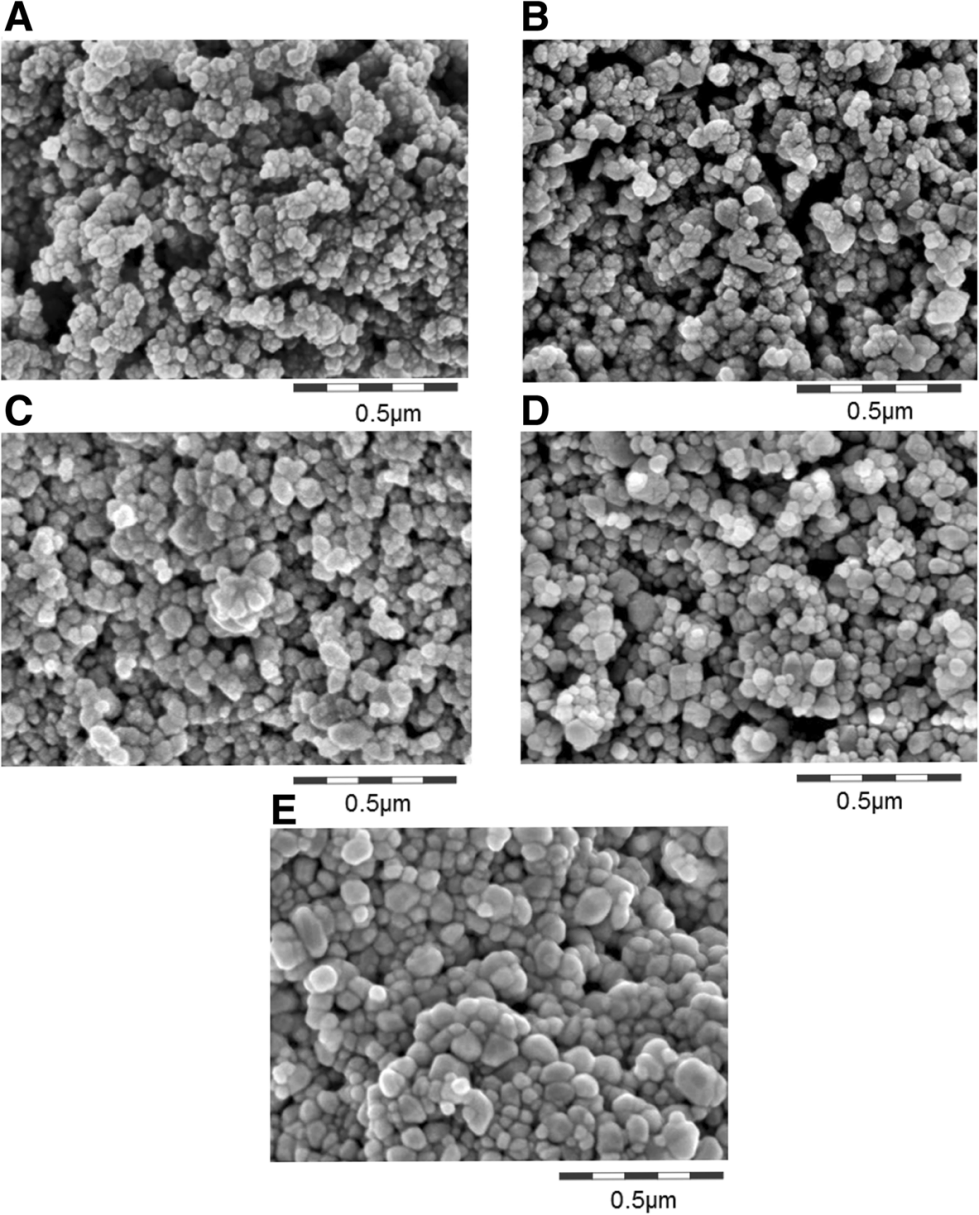
**Structural characterization by representative SEM scans of as-produced BaSO**_**4**_ **nanomaterial and after incubation for testing of persistence. (A)** reproduced batch, as-produced powder; **(B)** NM-220 batch, as-produced powder; **(C)** pellet of NM-220 after 28d incubation in PBS; **(D)** pellet of NM-220 after 28d incubation in PSF; **(E)** pellet of NM-220 after 1d incubation in 0.1 N HCl.

Barium sulfate NPs (NM-220) used in all IT instillation, gavage and IV injection studies were obtained from BASF SE (Ludwigshafen, Germany). This sample was a reference material for the Nanomaterial Testing Sponsorship Program of the OECD. The characterization of this original batch was published recently [29]. Since the chronic inhalation study requires large amounts (>100 kg), BaSO_4_ NPs were reproduced at a different production plant using the same synthesis protocol. This reproduced batch was characterized by the same methods and was used for the 4-week and 13-week inhalation studies. All physicochemical endpoints are summarized in Additional file 1: Table S1 (online Supporting Information), which includes the previously published characterization of NM-220 for comparison [29]. Transmission and scanning electron micrographs show that BaSO_4_ NPs in both batches were nonspherical globular with no fiber, rod or platelet impurities. The primary particle size was 25 nm for both batches (Figure 1A and 1B). The NPs form larger spherical agglomerates (2–15 μm diameter) in the as-produced powder (Additional file 1: Table S1). This agglomerate structure was confirmed by porosimetry which showed dominant pore sizes of 30 nm and 5 μm for both batches (Additional file 1: Figure S1A). X-ray diffraction (XRD) analysis showed that the particle mineralogy was orthorhombic barite (Additional file 1: Figure S1B) for both batches. Photocatalytic activity of both batches was extremely low as shown by the absence of methylene blue degradation (Additional file 1: Figure S2).

The shape, particle size distribution, primary particle diameter, state of agglomeration (powder), crystalline phase, specific surface area, surface charge, photocatalytic activity, and dispersability in water and in Dulbecco’s modified Eagle/fetal calf serum (DMEM/FCS) media were similar in both batches. Analyses by several methods (EM, minimal pore size, specific surface area) indicate that the two batches have similar primary particle sizes. The properties that were determined by surface chemistry such as dispersability, charge/zeta potential and photocatalytic reactivity were also similar (Additional file 1: Figure S2). However, significant differences were observed in crystallite size (36 nm for NM-220 vs. 23 nm for reproduced batch). The reproduced material also had an intermediate pore size of 200 nm (agglomerate structure) which was absent in the original NM-220 material. XPS analyses showed significantly less carbon atoms exposed on the surface of the reproduced material (2 vs. 17%). Additionally, elemental analysis by neutron activation showed that the NM-220 batch had 599 μg Ba/mg material (59.9 wt%), as expected for relatively pure BaSO_4_ (Table 1).

**Table 1:** **Neutron activation analysis of BaSO**_**4**_ **NM-220**

**Element**	**Concentration (μg/g)**
Ba	599,000 ± 33,000
Sr	9,820 ± 570
Na	3,022 ± 69
Zn	31.1 ± 2
Mn	0.41 ± 0.21
Co	0.164 ± 0.038
Au	0.04 ± 0.003
Sc	0.0206 ± 0.0055

Data are mean ± standard deviation.

In physiological simulant fluids, BaSO_4_ NPs (NM-220) dissolved only slightly at pH 1 (1% dissolution in 0.1 N HCl) although its particle shapes changed (Additional file 1: Table S1, Figure 1E) [29]. Very low (0.1%) dissolution was observed in phosphate buffered saline (PBS) or phagolysosomal simulant fluid (PSF, pH 4.5) after 28 days incubation (Additional file 1: Table S1). No morphologic changes were seen in PBS (Figure 1C). However, the non-spherical BaSO_4_ NPs lost their structural features with lowest radius of curvature and recrystallized to spherical structures in PSF (Figure 1D) [30]. It was confirmed by selected area electron diffraction that the crystallinity was retained (data not shown). BaSO_4_ NPs remained in a low agglomeration state and retained a significant dispersed fraction (80%) of ≤1 μm diameter in all simulant buffer conditions. The zeta potential ranged from −20 mV to −32 mV.

The agglomerate size of BaSO_4_ NPs in deionized water suspension employed in IT instillation (0.67 mg/ml), gavage (10 mg/ml) and IV injection (1 mg/ml) was assessed using dynamic light scattering (DLS). We found that BaSO_4_ NP agglomerate size was influenced by particle concentration (Table 2): the higher the concentration, the larger the hydrodynamic diameter. For the inhalation studies, the particle concentrations and size distributions are summarized in Table 3. The target concentration of 50 mg/m^3^ was achieved and maintained throughout the inhalation exposures. Particle size distribution of aerosolized BaSO_4_ NPs was in the respirable range for rats.

**Table 2:** **Dynamic light scattering analysis of BaSO**_**4**_ **NM-220 suspensions**

**Concentration (mg/ml dH**_**2**_ **O)**	**d**_**H**_ **(nm)**	**PdI**	**ζ (mV)**
0.66	144 ± 4	0.20 ± 0.03	−18.5
1	154 ± 21	0.24 ± 0.07	−18.7
10	354 ± 3	0.46 ± 0.03	−16.5

Data are mean ± standard deviation, n = 3.

d_H_, hydrodynamic diameter, PdI, polydispersity index, ζ, zeta potential.

**Table 3:** **Aerosol concentrations and particle size distributions of BaSO**_**4**_ **NM-220**

**Duration of exposure**	**Targeted concentrations (mg/m** ^**3**^ **)**	**Measured concentrations (mg/m** ^**3**^ **)**	**MMAD (μm)/GSD mean**	**Particle count concentration (particle/cm** ^**3**^ **)**	**Particle count median diameter (nm)**
4 weeks	50	46.2 ± 5.9	2.3/2.0	92752	326
13 weeks	50	50.1 ± 5.6	1.9/2.1	77992	304

#### Pulmonary responses to instilled or inhaled BaSO_4_ nanoparticles

To determine whether BaSO_4_ NPs elicit toxic or inflammatory response in rats and to identify a suitable dose for the IT biokinetic studies, groups of six rats were IT-instilled with BaSO_4_ NP suspension (NM-220) at 0, 1, 2, and 5 mg/kg body weight. We found that BaSO_4_ NPs caused an acute dose-dependent inflammatory response evidenced by significant increases in BAL parameters (Table 4). Neutrophils, myeloperoxidase (MPO) and lactate dehydrogenase (LDH) levels in bronchoalveolar lavage (BAL) were elevated 24 hours post-instillation. We also found that 2 and 5 mg/kg doses caused pulmonary hemorrhage and edema as indicated by increased BAL haemoglobin and albumin levels. Based on these data, we concluded that 1 mg/kg was the maximum safe dose for the IT biokinetic study, since injury and inflammation were minimal, yet it was sufficient for gamma detection of ^131^Ba in the lungs and other tissues over a period of 28 days.

**Table 4:** **Bronchoalveolar lavage analysis at 1 day after intratracheal instillation of BaSO**_**4**_ **NPs**

**Dose**	**Control**	**1 mg/kg**	**2 mg/kg**	**5 mg/kg**
BaSO_4_ lung burden (mg)	0	0.28 ± 0.004	0.56 ± 0.004	1.4 ± 0.004
Total Cells (million)	8.31 ± 1.01	12.59 ± 1.71	14.26 ± 0.74*	16.76 ± 1.15*
Macrophage (million)	8.31 ± 1.01	12.28 ± 1.62	11.41 ± 0.67	10.31 ± 1.40
Neutrophils (million	0.001 ± 0.00	0.29 ± 0.10	2.85 ± 0.29*	6.45 ± 1.09*
LDH (mU/ml)	38.10 ± 0.30	57.23 ± 4.98	171.52 ± 33.05	167.68 ± 18.53*
MPO (mU/ml)	0.94 ± 0.07	0.80 ± 0.21	26.48 ± 8.50	34.95 ± 8.82
Albumin (μg/ml)	3.28 ± 1.39	6.12 ± 0.38*	11.88 ± 1.20*	14.53 ± 1.12*
Hemoglobin (μg)	9.44 ± 1.39	16.56 ± 0.89*	24.79 ± 2.78*	37.24 ± 2.64*

Data are mean ± SE, n = 4-5/group.

*p <0.05, BaSO_4_ versus 0 mg/kg (Control, equivalent volume of distilled water).

To assess pulmonary responses of rats after short-term and subchronic inhalation of BaSO_4_ NPs, BAL analysis was performed one day (4- and 13-week groups) and 35 days (4-week group) after the end of each exposure protocol. Results for all BAL parameters are presented in Table 5. After 4 weeks of exposure, neutrophils were significantly increased compared to concurrent controls (filtered air-exposed) one day after the end of exposure. However, these values were within the historical control range in our previous studies. Rats exposed for 13 weeks showed significant increases in BAL total cells and neutrophils compared to control. These neutrophil counts were significantly lower than those seen in instilled rats (Table 4, Additional file 1: Figure S3). Cytokine levels of monocyte chemoattractant protein-1 (MCP-1) and cytokine-induced neutrophil chemoattractant-1 (CINC-1) were elevated in both exposure groups (Table 5). The longer 13-week exposure to BaSO_4_ NPs induced higher levels of the cytokine MCP-1 compared to the 4-week exposure. All BAL parameters elevated at 1 day post-exposure returned to control levels in the 4-week exposure group at 35 days. No morphological changes were detected by histopathology in the lungs (Additional file 1: Figure S4) and extrapulmonary organs. Other parameters such as body weights, micronucleus test of erythrocytes in peripheral blood, showed no significant change. Rats exposed for 13 weeks showed significantly higher gamma glutamyl transferase (GGT) and alkaline phosphatase (ALP) levels than their corresponding controls (Table 5).

**Table 5:** **Bronchoalveolar lavage analysis at 1 or 35 days after inhalation exposure to BaSO**_**4**_ **NPs**

	**4-week exposure**		**13-week exposure**	
	**Control**	**BaSO**_**4**_	**Control**	**BaSO**_**4**_
BaSO_4_ lung burden (mg)		0.84 ± 0.18		1.73 ± 0.85
Total cells (million)				
1 day	0.649 ± 0.20	0.562 ± 0.12	0.610 ± 0.20	0.836 ± 0.15*
35 days	0.580 ± 0.11	0.454 ± 0.12	ND	ND
Neutrophils (million)				
1 day	0.007 ± 0.003	0.021 ± 0.010*	0.016 ± 0.006	0.204 ± 0.175*
35 days	0.019 ± 0.008	0.032 ± 0.024	ND	ND
Total protein (mg/L)				
1 day	60 ± 4	77 ± 13*	52 ± 10	64 ± 13
35 days	81 ± 23	59 ± 13	ND	ND
GGT (nkat/L)				
1 day	37 ± 17	40 ± 10	41 ± 7	64 ± 13*
35 days	42 ± 12	37 ± 12	ND	ND
ALP (μkat/L)				
1 day	0.83 ± 0.16	0.83 ± 0.20	0.51 ± 0.10	0.87 ± 0.21*
35 days	0.70 ± 0.09	0.70 ± 0.12	ND	ND
MCP-1 (pg/ml)				
1 day	14.0 ± 0.0	54.7 ± 14.3*	24.2 ± 8.4	176.7 ± 126.1*^#^
35 days	17.3 ± 2.6	14.7 ± 1.7	ND	ND
CINC-1/IL-8 (pg/ml)				
1 day	104.2 ± 26.7	158.7 ± 22.4*	93.7 ± 18.7	223.8 ± 125.7*
35 days	158.8 ± 38.1	167.6 ± 41.1	ND	ND

Data are mean ± SD, n = 5/group. Control rats were exposed to filtered air.

ND, not determined.

*p ≤0.05, BaSO_4_-exposed vs. control; ^#^p ≤0.05, 13-week vs. 4-week exposure.

Neutrophils counts were significantly much lower compared to data from rats instilled with 1.4 mg BaSO_4_ (5 mg/kg BaSO_4_) (Table 4).

### In vivo clearance and translocation of ^131^BaSO_4_ nanoparticles after IT instillation in rats

**Figure 2:**
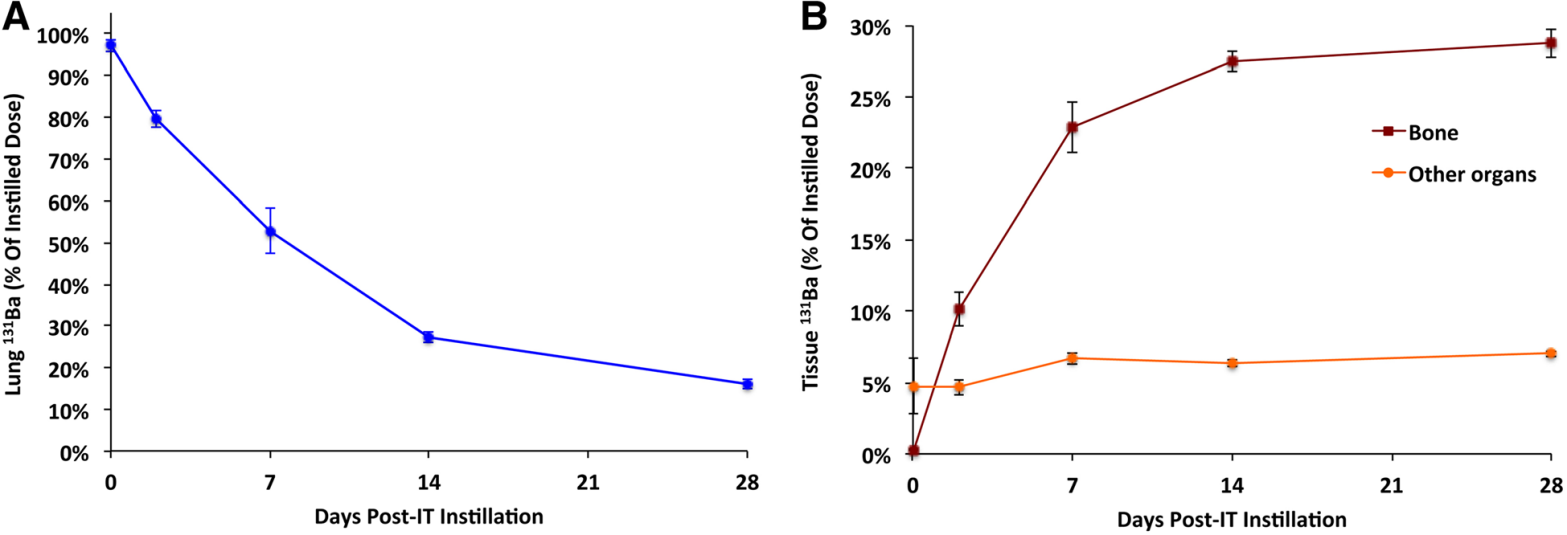
**Clearance and translocation of** ^**131**^ **BaSO**_**4**_ **NPs following IT instillation. (A)** Lung clearance of ^131^BaSO_4_ over time. The clearance half life was approximately 9.6 days. By 28 days, 84% of dose has been cleared from the lungs. **(B)** Translocated ^131^Ba from the lungs gradually accumulated in other organs. By 28 days, 29% of the instilled ^131^Ba dose was retained in the bone and 7% in all the other organs. Data are mean ± standard error of the mean, n = 5 per group.

**Figure 3:**
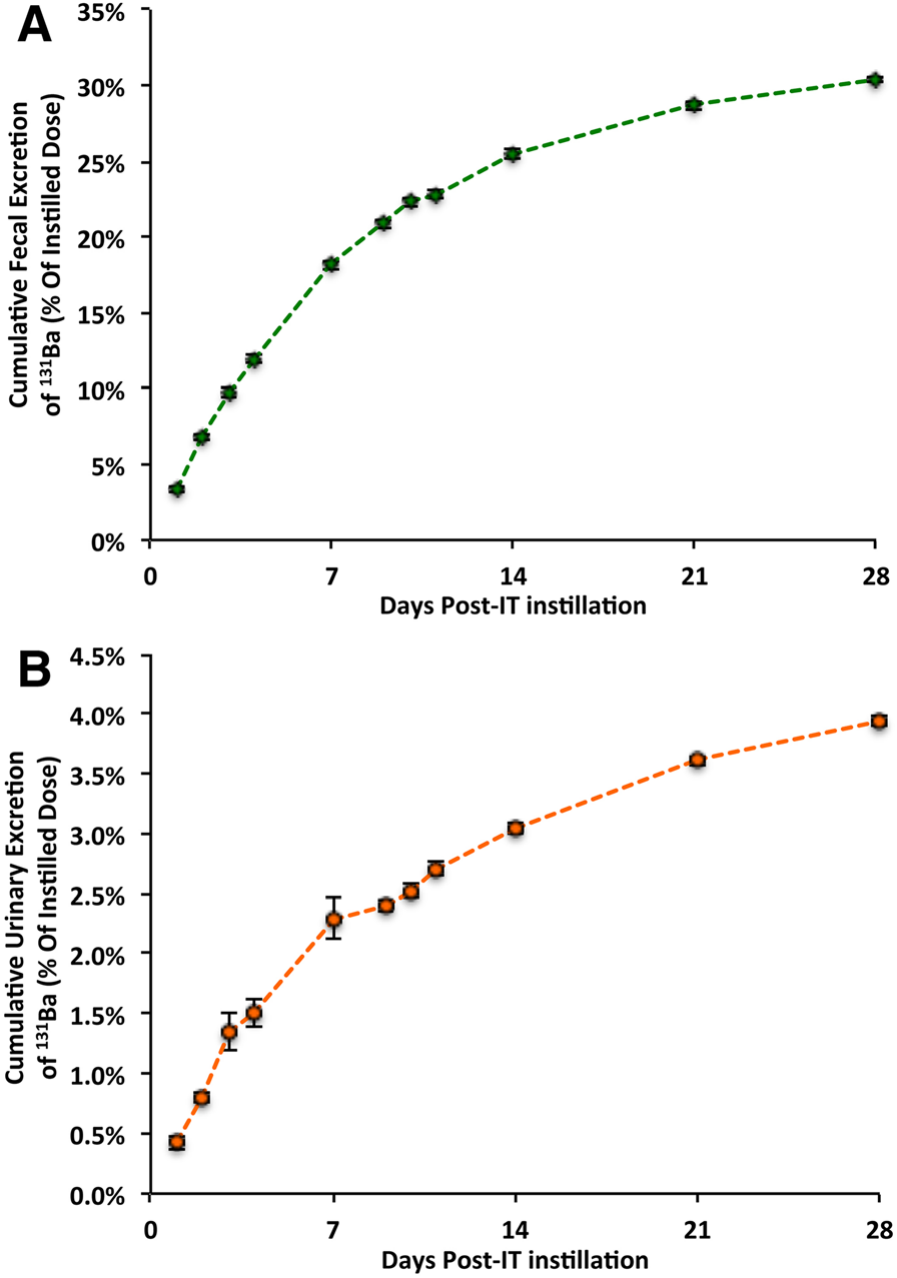
**Cumulative fecal and urinary excretion of** ^**131**^ **Ba following IT instillation.** Elimination of ^131^Ba was mainly via the feces. By 28 days post-dosing, 30% of the instilled dose was excreted in the feces **(A)** and only 4.4% in the urine **(B)**. Data are mean ± standard error of the mean, n = 5 per group.

The clearance of ^131^BaSO_4_ NPs from the lungs post-instillation is shown in Figure 2A. Approximately 47% of the total dose was cleared from the lungs by day 7 and 84% by day 28. A linear regression on the natural logarithm of the lung ^131^BaSO_4_ levels (% dose) over time was performed (y = e^-0.003011x^ , R^2^ = 0.96, p = <0.0001). The estimated clearance half-life was 9.6 days. Extrapulmonary translocation of ^131^Ba is shown in Figure 2B. A significant fraction of ^131^Ba radioactivity was found in the bones (29% of dose) and lower fractions in all other tissues combined (7%). The rest of the ^131^Ba was excreted mostly in the feces (30%) and to a lesser extent in the urine (3.9%) (Figure 3). The complete distribution data of ^131^Ba after instillation of ^131^BaSO_4_ are summarized in Additional file 1: Table S2.

### Fate of ^131^BaSO_4_ nanoparticles after oral administration in rats

**Figure 4:**
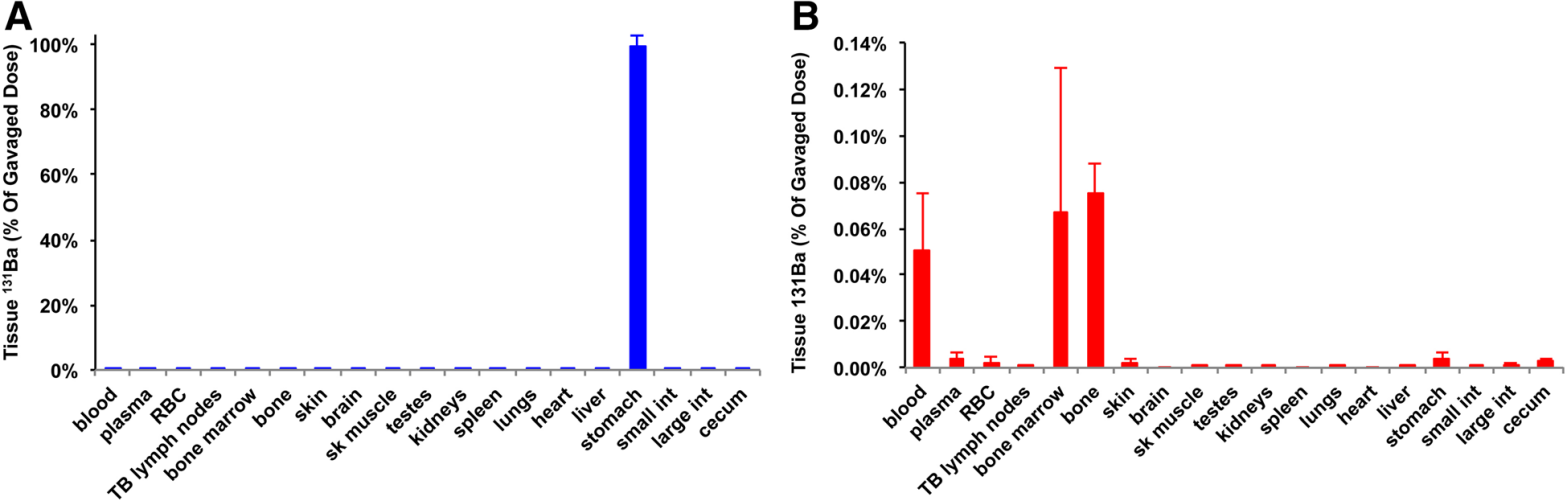
**Tissue distribution of** ^**131**^ **Ba following gavage. A**. Immediately post-gavage, 100% of dose was recovered in the stomach. **B**. At 7 days post-gavage, ^131^Ba was negligible in all tissues. Very low percentages of the dose were detected in bone and bone marrow. Data are mean ± standard error of the mean, n = 5 per group.

**Figure 5:**
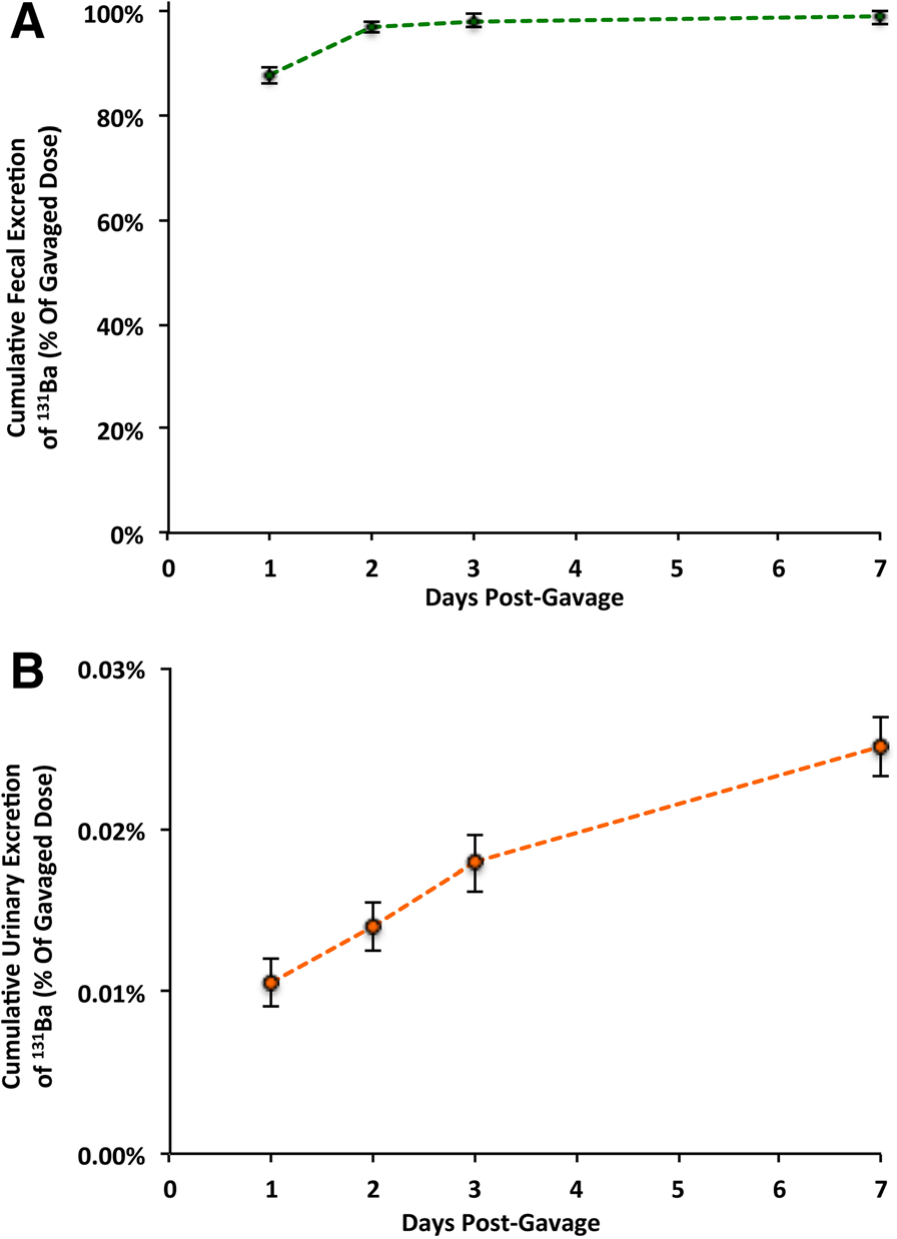
**Cumulative fecal and urinary excretion of** ^**131**^ **Ba post-gavage. A**. Elimination of ^131^Ba was nearly 100% via the feces. **B**. By 7 days post-gavage, only 0.02% of the administered dose was excreted in the urine. Data are mean ± standard error of the mean, n = 5 per group.

The tissue distribution of ^131^Ba activity following oral administration is summarized in Figure 4 and listed in Additional file 1: Table S3. Nearly 100% of the administered dose was measured in the stomach at 5 minutes post-gavage (Figure 4A). At 7 days, very low percentages of the total dose were detected in blood, bone and bone marrow (<0.1%) (Figure 4B). Gavaged ^131^BaSO_4_ NPs were mostly cleared from the GI tract and eliminated in the feces (Figure 5A). Only 0.02% was excreted in the urine (Figure 5B).

### Tissue distribution of ^131^BaSO_4_ nanoparticles after intravenous injection in rats

**Figure 6:**
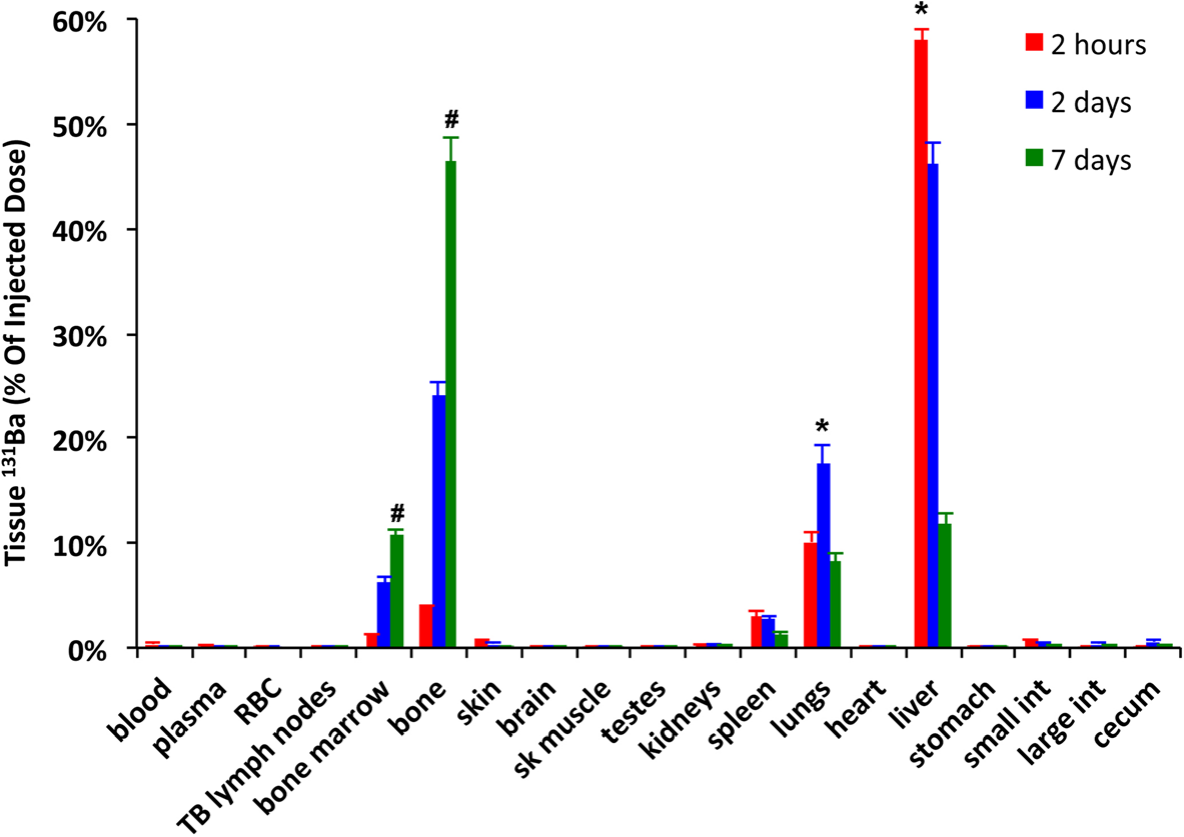
**Tissue distribution of** ^**131**^ **Ba post-IV injection.** Two hours post-injection, 58% of the injected dose was recovered in the liver and lower percentages in the spleen, bone, bone marrow and the lungs. Over time, ^131^Ba levels in the liver and lungs decreased with accompanying increases in bone and bone marrow. Data are mean ± standard error of the mean, n = 5 per group. *P <0.05, decrease over time, ^#^P <0.05, increase over time, MANOVA).

At 2 hours after intravenous injection of ^131^BaSO_4_ NPs, the blood levels of ^131^Ba were less than 0.5% of the administered dose (Figure 6). The complete distribution data at various time point post-injection are summarized in Additional file 1: Table S4. The tissue distribution was typical of circulating particles that are taken up in organs comprising the mononuclear phagocyte system with access to the circulation [31]. Notably, ^131^BaSO_4_ NPs were predominantly localized in the liver, spleen, bone and bone marrow. Interestingly, a significant fraction was also measured in the lungs. This may represent the larger agglomerates that may be lodged within pulmonary capillaries. Over the period of 7 days after IV administration, ^131^Ba in the liver significantly decreased and redistributed into lungs, bone, and bone marrow (Figure 6). ^131^Ba activity in the lungs also significantly decreased over time (Figure 6). By day 7, a significant fraction of ^131^Ba radioactivity was found in the bones (46%). The cumulative fecal and urinary excretions of ^131^Ba are shown in Additional file 1: Figure S5. The cumulative fecal excretion was 17% while only 4% of the total injected dose was excreted in the urine over a period of 7 days (Additional file 1: Figure S5B).

### Barium tissue concentration - influence of route of exposure

We examined how the route of exposure affects tissue barium concentrations after dosing with ^131^BaSO_4_ NPs. Using the measured specific activity of ^131^BaSO_4_ NPs and each tissue ^131^Ba concentration, we estimated Ba concentration in ng Ba/g tissue. The Ba concentrations at 7 days post-dosing are shown in Table 6. These data demonstrate that IT instillation resulted in significantly higher tissue concentrations than gavage, especially in the bone. Barium tissue levels ranged from very low to not detectable post-gavage despite dosing the animals with a higher mass dose (1 v. 5 mg/kg). As expected, IV injection resulted in higher Ba concentrations in most tissues compared to IT and gavage administration.

**Table 6:** **Comparison of tissue barium concentrations at 7 days after dosing**

**Route (dose)**	**IT Instillation (1 mg/kg)**	**Gavage (5 mg/kg)**	**IV Injection (1 mg/kg)**
	**ng/g ± SE**	**ng/g ± SE**	**ng/g ± SE**
Lungs	69074.5 ± 4993.8	< 0.01	9847.5 ± 983.8
Bone	2271.9 ± 124.6*	0.079 ± 0.014	3788.5 ± 156.6^**#**^
Bone marrow	1018.3 ± 71.4*	0.13 ± 0.12	1641.3 ± 62.2^**#**^
Cecum	119.3 ± 23.8	< 0.01	61.2 ± 4.2^**#**^
TB LN	113.1 ± 16.3	< 0.01	64.8 ± 14.1
Large Intestine	106.6 ± 31.2	< 0.01	72.8 ± 6.1^**#**^
Small Intestine	43.2 ± 6.7	< 0.01	28.5 ± 2.3
Spleen	37.5 ± 1.5	< 0.01	3256.4 ± 391.2^**#**^
Stomach	30.3 ± 18.6*	0.016 ± 0.016	46.3 ± 10.1
Kidneys	7.8 ± 2.7	< 0.01	140.8 ± 12.9^**#**^
Plasma	3.6 ± 1.2	< 0.01	0.1 ± 0.1
Heart	3.6 ± 1.2	< 0.01	77.5 ± 10.8^**#**^
Brain	3.5 ± 0.8	< 0.01	18.6 ± 4.5^**#**^
RBC	3.1 ± 0.5	< 0.01	< 0.01
Skeletal Muscle	3.0 ± 0.8	< 0.01	8.3 ± 0.5^**#**^
Liver	2.5 ± 0.5	< 0.01	1760.0 ± 221.9^**#**^
Skin	2.5 ± 1.0	< 0.01	2.5 ± 0.8
Testes	0.7 ± 0.7	< 0.01	3.3 ± 3.3

Data are mean ± SE, n = 5/group.

*P <0.05, IT instillation higher than gavage. All other tissues post-gavage were lower than detection limit ~0.01 ng/g Barium.

^#^P <0.05, IV injection higher than IT instillation.

TB LN, tracheobronchial lymph nodes.

### Lung and lymph node barium analysis after inhalation exposure to BaSO_4_ nanoparticles

The amounts of BaSO_4_ in the lungs and lymph nodes were estimated by measuring Ba with ICP-MS. Inhalation exposure to 50 mg/m^3^ resulted in an equivalent BaSO_4_ lung burden of 0.84 ± 0.18 mg at 1 day after the end of a 4-week exposure. Lung BaSO_4_ burden decreased by 95% (0.84 ± 0.18 to 0.04 mg) on day 1 versus day 35 after exposure. After 13 weeks of exposure, the lung, tracheobronchial and mediastinal lymph node burdens were 1.73 ± 0.85 mg, 5.92 ± 6.52 μg, 2.72 ± 3.38 μg BaSO_4_, respectively.

## Discussion

Our studies examined the effects of short-term (4-week) and subchronic (13-week) inhalation exposure and a single IT instillation of BaSO_4_ NPs in rats. We also performed comprehensive biokinetic studies of ^141^Ba when ^141^BaSO_4_ NPs were administered in rats via different routes. Four weeks of inhalation of 50 mg/m^3^ BaSO_4_ resulted in no pulmonary toxicity by 35 days post-exposure. BAL parameters were comparable to control values after the post-exposure period. Delayed onset of adverse effects beyond this post-exposure period is unknown. Histopathologic examination performed in 4-week exposed animals showed no morphological changes in lungs and extrapulmonary organs (e.g. brain, heart, liver, spleen, kidneys). These results are consistent with our previous short-term inhalation study that tested a variety of nanomaterials including BaSO_4_ NPs [27]. A 13-week exposure elicited a slight inflammatory response in rat lungs. The long-term effects of inhalation exposure to BaSO_4_ NPs are being evaluated in an ongoing two-year study. Our instillation data showed a moderate dose-dependent inflammatory response to BaSO_4_ NPs at 24 hours. Lung burdens at 1 day after 4 or 13 weeks of inhalation exposure were 0.84 ± 0.18 and 1.73 ± 0.85 mg BaSO_4_/lung, respectively. At the 5 mg/kg instilled dose (1.4 mg BaSO_4_ lung burden) the neutrophil response was significantly higher than at 24 hours after the last inhalation exposure. The difference in neutrophil response may be due to the differences in dose rate, particle distribution, particle clearance, agglomerate surface properties and gender between the two studies. That the two exposure methods yield different responses is also consistent with previous reports [32,33]. A study by Baisch et al. reported a higher inflammatory response to a similar deposited dose of TiO_2_ NPs when delivered via IT instillation rather than whole body inhalation [32]. It is clear that although IT instillation is a reliable method for administering a precise dose to the lungs, it does not model inhalation exposure. Particle distribution and dose rate are different between these two exposure methods. However, IT instillation is useful in biokinetic studies that require precise dosing and timing especially for radioactive materials such as ^131^BaSO_4_ NPs. Our use of radiolabelled NPs provided a very sensitive method that measured only ^131^Ba from the nanoparticles and excluded background Ba from other sources, such as food and water. The sensitivity of ^131^Ba detection also avoided the use of high BaSO_4_ doses while allowing us to measure very low levels in tissues.

Pulmonary clearance kinetics post-inhalation was similar to the previous 5-day inhalation study [28]. We observed a 95% clearance of Ba from the lungs in 34 days. This is consistent with the previously observed 77% clearance over 21 days [28]. The lung burden of BaSO_4_ after 13-week exposure to a high concentration of BaSO_4_ was also similar to those of rats exposed to lower concentrations of TiO_2_ and CeO_2_ [28,34]. This shows that clearance of BaSO_4_ is much faster than these other two nanomaterials. But the similar lung burdens from exposure to TiO_2_ and CeO_2_ resulted in greater inflammatory responses [10,28]. Our data suggest that the low toxicity of inhaled BaSO_4_ is inherent to the nanomaterial as well as its relatively faster clearance.

The biokinetic data based on radioactive ^131^BaSO_4_ showed a fast clearance of ^131^Ba from the lungs. We observed that 50% of the initial dose was cleared from the lungs after 9.6 days. By 28 days only 16% of the initial dose was retained in the lungs. This clearance rate also roughly correlated with that obtained from our inhalation experiment. Based on a linear regression on the natural logarithm of lung ^131^Ba levels (% of instilled dose) over time, the extrapolated clearance of instilled ^131^BaSO_4_ dose at 35 days is 92%. The lung burden post-instillation was 0.28 ± 0.004 which was lower than lung burden after 4-week inhalation (0.84 ± 0.18 mg BaSO_4_). Despite this difference in initial lung burden, the clearance rate of BaSO_4_ NPs was not different between the two exposure methods.

The lung clearance of BaSO_4_ NPs was similar to that shown for micron-sized radiolabeled BaSO_4_ where 17% of radiolabeled barium remained at 22 days post-instillation in rat lungs [20,21]. The fate of the 16% of ^131^Ba remaining in the lungs at the end of our observation needs longer-term studies. Since lung epithelial injury may alter the fate of instilled NPs, we chose a dose that would not cause significant injury that might affect the outcome of our IT biokinetic study. Our data suggest that ^131^Ba from instilled ^131^BaSO_4_ NPs was cleared from the entire animal mainly via the gastrointestinal route. The excreted fraction in the feces might include contributions from both the mucociliary and biliary clearance pathways. Although lung clearance of ^131^BaSO_4_ NPs is relatively fast and only 16% of the administered dose remained 4 weeks post-instillation, a substantial fraction (37%) was retained elsewhere in the body. The tissue distribution of ^131^Ba following IT instillation showed a significant translocation to bone, consistent with other heavy earth alkaline metals like calcium and strontium [35] as well as to the thoracic lymph nodes. Whether the ^131^Ba measured in these extrapulmonary organs was ^131^BaSO_4_ NPs or ionic ^131^Ba could not be ascertained in this study. A previous study showed that IT-instilled ionic barium cleared much more rapidly than BaSO_4_ particles [36]. We have also demonstrated that ionic cerium was more toxic and was cleared more rapidly than CeO_2_ NPs [37].

The clearance of inhaled BaSO_4_ NPs was fast as evidenced by the decrease in BaSO_4_ lung burden over time. Only 5% of retained BaSO_4_ in the lungs (4-week-exposure) remained 35 days after the end of exposure. The relatively high bioavailability of inhaled or instilled BaSO_4_ does not correlate with its very low dissolution rate in phagolysosomal simulant fluid, a proposed model of macrophage dissolution/clearance of particles [30]. This strongly suggests that PSF does not fully simulate the complex kinetic processes of lung transport and clearance, especially the mechanism of particle dissolution within macrophage phagolysosomes. Our cell-free *in vitro* dissolution studies showed very low dissolution in PSF even after 28 days. However, we observed that the non-spherical BaSO_4_ NPs lost their feature of lowest radius of curvature and later recrystallized over this period. Interestingly, it has been shown that the NP surface charge and interactive properties may vary with the local radius of curvature [38]. The regions of the particle surface with different curvature become charged at differing pH values of the surrounding solution [38]. Previous studies showed that non-spherical nanomaterial may exhibit different toxicity from that of spherically shaped nanomaterial of the same composition due to the varying local charge density [39]. Likewise, quartz and vitreous silica NPs, with irregular surfaces and sharp edges were more toxic than spherical silica [40]. The significance of the noted structural changes of BaSO_4_ NPs *in vitro* remains to be studied in the phagolysosomal compartment of lung macrophages. How these structural changes relate to cytotoxicity is likewise yet to be determined.

Whole-body exposure of rats to NP aerosols results in not only pulmonary deposition but also in ingestion of NPs due to the grooming behavior of rats. This ingestion can complicate the pattern of bioavailability from whole-body inhalation exposures. However, for animal welfare considerations, whole-body is more convenient than nose-only exposure for long-term inhalation studies. For some applications of BaSO_4_, the gastrointestinal tract is also a common route for human exposures. Thus, we investigated the fate of orally administered ^131^BaSO_4_ NPs. Since the GI transit time is generally less than one day, it is less likely for nanoparticles to remain in the GI tract for a prolonged period of time. Even particles adherent to or ingested by columnar epithelial cells are eliminated rapidly since the epithelium sloughs off and regenerates constantly. Our data showed that 88% of the dose was eliminated in the feces within 24 hours, and almost 100% by 7 days post-gavage. Since very low radioactivity was detected in other organs, we conclude that neither ^131^BaSO_4_ NPs nor ^131^Ba ions significantly crossed the intestinal barrier. This low oral bioavailability correlates with our observation of very low dissolution of BaSO_4_ NPs in simulated gastric and intestinal fluid (Additional file 1: Table S1). This indicates that there is negligible contribution from fur deposition and ingestion during inhalation exposure. It also means that barium detected in extrapulmonary organs after inhalation translocates from the lungs to the blood.

As we and others have shown, a small fraction of inhaled nanoparticles may translocate into the systemic circulation [41]. Although our study focused on normal lungs, when they are compromised by injury or inflammation increased rates of NP translocation may occur. Therefore, we evaluated the biokinetics and tissue distribution of intravenously injected ^131^BaSO_4_ NPs to elucidate their fate in the circulation. When we sacrificed animals at 2 hours post-IV injection of ^131^BaSO_4_ NPs, we found very low radioactivity in the blood. Since the first time point we examined was at 2 hours, we could not determine the vascular clearance rate. Previously, we have shown that clearance half-lives of circulating particles are on the order of minutes even for nanoparticulates such as gold colloid [31]. Initially, a significant hepatic accumulation of ^131^Ba was observed but liver retention was decreased by 7 days. This likely reflects rapid ingestion of ^131^BaSO_4_ NPs by the abundant hepatic macrophages (Kupffer cells) and possibly subsequent dissolution followed by release of barium ions into the blood. The decrease was accompanied by increasing accumulation in bone similar to that observed following IT instillation. Despite the significant uptake of Ba in the bone, no evidence of genotoxicity in the bone marrow was noted. We found no micronucleus formation in peripherial blood cells that originate from hematopoiesis in the bone marrow.

## Conclusions

Our data show that inhaled BaSO_4_ NPs elicited minimal pulmonary response and no systemic effects. Equivalent lung burdens of CeO_2_ and TiO_2_ elicit more pulmonary response than BaSO_4_ [28]. This difference might be due to its lower inherent toxicity and also to its faster lung clearance. The mechanism of this faster clearance needs further investigation. There is no direct correlation between abiotic *in vitro* dissolution of BaSO_4_ in several cell-free biological simulation fluids and actual *in vivo* biopersistence and bioavailability of barium from BaSO_4_ NPs. Our data suggest that cell-free *in vitro* assays either lack crucial constituents or do not adequately simulate the processes that facilitate particle dissolution and increase bioavailability. The Ba in BaSO_4_ from the lungs translocates to many tissues, especially the bones. The comparison of pulmonary versus ingestion routes of exposure provides a quantitative measure of relative doses to a variety of non-pulmonary tissues. From our data, it is evident that the bioavailability of Ba from ingestion of BaSO_4_ NPs is very low and that no significant contribution from ingestion should occur during whole-body inhalation studies in rats.

Our study underscores the high Ba bioavailability and clearance of BaSO_4_ NPs deposited in the lungs. Unlike CeO_2_ and TiO_2_, BaSO_4_ NPs are retained to a lesser extent in the lungs after inhalation. Even at lung burdens similar to CeO_2_ and TiO_2_, BaSO_4_ NPs cause lower pulmonary toxicity. Barium sulfate exhibits lower toxicity and biopersistence in the lungs compared to poorly soluble CeO_2_ and TiO_2_.

## Methods

### Physicochemical characterization of BaSO_4_ nanoparticles

BaSO_4_ NPs (NM-220) used for IT instillation studies were obtained from BASF SE (Ludwigshafen, Germany). It was a reference material for the Nanomaterial Testing Sponsorship Program of the Organization for Economic Cooperation and Development (OECD). The characterization of the original batch distributed as “NM-220” was published recently [29]. The reproduced BaSO_4_ used in inhalation studies was characterized by the same methods (See Supporting Information).

### Animals for intratracheal instillation, gavage and intravenous injection studies

The protocols used in this study were approved by the Harvard Medical Area Animal Care and Use Committee. Male Wistar rats (8 weeks old) were obtained from Charles River Laboratories (Wilmington, MA) and were housed in standard microisolator cages under controlled conditions of temperature, humidity, and light at the Harvard Center for Comparative Medicine. They were fed commercial chow (PicoLab Rodent Diet 5053, Framingham, MA) and reverse-osmosis purified water was provided *ad libitum*. The animals were acclimatized in the facility for 7 days before the start of experiments.

### Preparation of BaSO_4_ suspension for animal dosing

Suspensions of BaSO_4_ NPs were prepared at appropriate concentrations in sterile polyethylene tubes. The critical dispersion sonication energy (DSE_cr_) required to achieve the lowest reported particle agglomeration was used as previously reported [42]. Suspensions in sample tubes were sonicated with a Branson Sonifier S-450A (Branson Ultrasonics, Danbury, CT) fitted with a cup sonicator at 242 J/ml, the critical dispersive energy shown to maximally disperse these particles in water [42] while immersed in running cold water to minimize heating of the particles. The hydrodynamic diameter (d_H_), polydispersity index (PdI), and zeta potential (ζ) of each suspension were measured by dynamic light scattering using a Zetasizer Nano-ZS (Malvern Instruments, Worcestershire, UK).

### Pulmonary responses to intratracheally instilled BaSO_4_ nanoparticles – Bronchoalveolar lavage and analyses

This experiment was performed to determine a particle dose for pulmonary particle instillation that does not cause significant injury or inflammation. Twenty rats (mean wt ± standard deviation, 280 ± 15 g) were IT-instilled with BaSO_4_ suspension at 1, 2 and 5 mg/kg dose (5 rats per dose) to determine the acute pulmonary effects of BaSO_4_ particles. The nanoparticle concentrations were 0.67, 1.33, and 3.33 mg/ml for the 1, 2, and 5 mg/kg dose, respectively. Rats instilled with an equivalent volume of sterile distilled water served as controls. The volume dose was 1.5 ml/kg. The particle suspensions were delivered to the lungs through the trachea, as described earlier [43]. Twenty-four hours later, the rats were anesthetized and euthanized via exsanguination. The trachea was exposed and cannulated. The lungs were then lavaged 12 times with 3 mL of Ca- and Mg-free 0.9% sterile PBS. The cells of all washes were separated from the supernatant by centrifugation (350 × g at 4°C for 10 min). Total cell count and hemoglobin measurements were made from the cell pellets. After smearing and staining the cells, a differential cell count was performed. The supernatant of the two first washes was clarified via centrifugation (14,500 × g at 4°C for 30 min), and used for spectrophotometric assays for lactate dehydrogenase (LDH), myeloperoxidase (MPO) and albumin.

### Neutron activation of BaSO_4_ nanoparticles for pharmacokinetic studies

Barium sulfate NM-220 particles were neutron activated at the MIT Nuclear Reactor Laboratory (Cambridge, MA) with a thermal neutron flux of 5 × 10^13^ n/cm^2^s for 24 hours. The process generated ^131^Ba, which decays with a half life of 10.5 days and emits multiple gamma rays with varying energies. The specific activity was 2.6 μCi ^131^Ba per mg BaSO_4_ NPs.

#### Pharmacokinetics of tracheally-instilled, gavaged or intravenously-injected ^131^BaSO_4_ nanoparticles

Fifty rats (mean wt ± standard deviation, 270 ± 12 g) were used for this study. Neutron-activated ^131^BaSO_4_ NPs were suspended in sterile distilled water at 0.67 mg/ml for intratracheal instillation (IT), 10 mg/ml for gavage, and 1 mg/ml for intravenous (IV) injection. The mass and volume doses were 1) IT - 1 mg/kg (1.5 ml/kg), 2) gavage - 5 mg/kg (0.5 ml/kg), and 3) IV - 1 mg/kg (1 ml/kg). The particle suspensions were dispersed as described earlier. Aliquots of each suspension were measured in a WIZARD gamma counter (PerkinElmer, Inc., Waltham, MA) to estimate each rat’s ^131^Ba dose. Gamma energies at 200–270 KeV were utilized for ^131^Ba quantitation. Each rat was anesthetized with isoflurane (Piramal Healthcare, Bethlehem, PA) during particle administration. After dosing, each rat was placed in a metabolic cage with food and water *ad libitum*. Twenty-four-hour samples of urine and feces were collected at 0–1, 2–3, 6–7, 9–10, 13–14, 20–21, and 27–28 days after dosing.

The ^131^BaSO_4_ NP suspension was delivered to the lungs through the trachea as described earlier. For gavage, ^131^BaSO_4_ NPs were delivered into the stomach via the esophagus. IV injection was done using the penile vein in similarly anesthetized animals. Five rats from the IT group were humanely killed at each time point: 5 minutes and 2, 7, 14 and 28 days post-dosing. Analysis of rats at 5 minutes post-instillation was performed to get an accurate measure of the initial deposited dose. Equal numbers of rats (5 per timepoint) were analyzed at 5 minutes and 7 days post-gavage, and at 2 hours, 2 days, and 7 days post-IV injection. At each time point, rats were anesthetized and blood collected from the abdominal aorta. Plasma and red blood cells were separated by centrifugation. The lungs, brain, heart, spleen, kidneys, gastrointestinal tract, liver, testes, and samples of skeletal muscle, bone marrow, skin, and femoral bone were collected and placed in pre-weighed tubes. Sample weight was recorded and radioactivity (200–270 KeV) was measured in a WIZARD gamma counter (PerkinElmer, Inc., Waltham, MA). Disintegrations per minute were calculated from the counts per minute and the counter efficiency. The limit of detection for ^131^Ba was 0.05 nCi. All radioactivity data were adjusted for physical decay over the entire observation period. Data were expressed as μCi/g and as a percentage of the administered dose retained in each organ. Total radioactivity in organs and tissues not measured in their entirety was computed using the following estimates of tissue percentage of total body weight: skeletal muscle, 40%; bone marrow, 3.2%; peripheral blood, 7%; skin, 19%; and bone, 6% [44,45].

### Animals for inhalation studies

Protocols for the inhalation studies were approved by the local authorizing agency in Landesuntersuchungsamt Koblenz, Germany. Animals were housed in an AAALAC-accredited facility in accordance with the German Animal Welfare Act and the effective European Council Directive. Female Wistar Han rats were obtained at 5 or 7 weeks of age from Charles River Laboratories (Sulzfeld, Germany). The animals were maintained in groups of up to 5 animals in a polysulfon cage (H-Temp [PSU], TECNIPLAST, Germany) with a floor area of about 2065 cm^2^ with access to wooden gnawing blocks, GLP certified diet (Kliba laboratory diet, Provimi Kliba SA, Kaiseraugst, Basel Switzerland) and water *ad libitum*. Animal rooms were kept under controlled conditions (20 - 24°C temperature, 30-70% relative humidity, 15 air changes per hour, 12-hour light/dark cycle). To adapt to the exposure conditions, the animals were acclimatized to exposure conditions over two days (3 and 6 hours, respectively). Up to two animals per wire cage type DK III (BECKER & Co., Castrop-Rauxel, Germany) were exposed in the whole-body exposure chamber.

### Study design - inhalation exposure for four and thirteen weeks

Thirty female rats (in groups of five) were whole-body exposed to 50 mg/m^3^ BaSO_4_ NPs for 6 hours per day on five consecutive days for 4 weeks (15 rats). Another cohort of 15 rats was exposed for 13 weeks. Body weights were recorded before and every week throughout the duration of the experiments. After 4 weeks of exposure, one group was examined and another after a post-exposure period of 35 days. The short-term inhalation study with 4 weeks of exposure was performed according to the OECD Principles of Good Laboratory Practice (GLP) [46], according to OECD Guidelines for Testing of Chemicals, Section 4: Health Effects, No. 412 [47]. This study provides information on biokinetics and effects of BaSO_4_ NPs required for the design of the long-term inhalation study. Barium burden in lungs was measured at three time points to determine the retention half-life. BAL analysis and histopathology of the lungs were performed. In addition, systemic effects were investigated with histopathology of extrapulmonary organs, examination of blood and systemic genotoxicity by micronucleus test (MNT). Based on the result of the short-term study with 4 weeks of exposure, the long-term study was started at the same concentration of 50 mg/m^3^ BaSO_4_. The long-term inhalation study is performed according to OECD Guidelines for Testing of Chemicals, Section 4: Health Effects, No. 453 [48].

#### Inhalation system

The animals were exposed while in wire cages that were located in a stainless-steel whole-body inhalation chamber (V = 2.8 m^3^ or V = 1.4 m^3^). The aerosols were passed into the inhalation chambers with the supply air and were removed by an exhaust air system with 20 air changes per hour. For the control animals, the exhaust air system was adjusted in such a way that the amount of exhaust air was lower than the filtered clean, supply air (positive pressure) to ensure that no laboratory room air reaches the control animals. For the BaSO_4_-exposed rats, the amount of exhaust air was higher than the supply air (negative pressure) to prevent contamination of the laboratory as a result of potential leakages from the inhalation chambers.

### Aerosol generation and monitoring

BaSO_4_ aerosols were produced by dry dispersion of powder pellets with a brush dust generator using compressed air at 1.5 m^3^/h (developed by the Technical University of Karlsruhe in cooperation with BASF, Germany). The dust aerosol was diluted by conditioned air passed into the whole-body inhalation chambers. The control group was exposed to conditioned clean air. The desired concentrations were achieved by varying the feeding speed of the powder pellet or by varying the rotation speed of the brush. Based on a comprehensive technical trial, atmospheric concentrations within the chambers were found to be homogenous (Table 3). Nevertheless, exposure cages were rotated within each chamber daily for the 4-week, and weekly for the 13-week group.

Generated aerosols were continuously monitored by scattered light photometers (VisGuard, Sigrist). Particle concentrations in the inhalation chambers were analyzed by gravimetric measurement of air filter samples. Particle size distribution was determined gravimetrically by cascade impactor analysis using eight stages Marple Personal Cascade Impactor (Sierra-Anderson, USA). In addition, a light-scattering aerosol spectrometer (WELAS 2000, Palas, Karlsruhe, Germany) was used to measure particle sizes from 0.24 to 10 μm. To measure particles in the submicrometer range, a scanning mobility particle sizer (SMPS 5.400, Grimm Aerosoltechnik, Ainring, Germany) was used. The sampling procedures and measurements to characterize the generated aerosols were previously described [49].

### Pulmonary responses to inhaled BaSO_4_ nanoparticles - Bronchoalveloar lavage and analysis

Five animals per group were examined. After euthanasia, the lungs were lavaged twice *in situ* with 22 mL/kg body weight (4 to 5 ml) of normal saline. The recovered volume ranged from 8 to 10 ml per animal. Aliquots of BAL were used for determinations of total protein concentration, total cell count, differential cell count and enzyme activities. In the 4 week-exposure group and its control, BAL analysis was performed twice (1 and 35 days after the end of exposure) but only at 1 day post-exposure in the 13-week exposure group. Lavaged lung tissue and aliquots of the BAL fluid (1 ml) were stored at −80°C and used for determination of barium content. Total BAL cell counts were determined with an Advia 120 (Siemens Diagnostics, Fernwald, Germany) hematology analyzer. Differential cell counts were made on Wright-stained cytocentrifuge slide preparations. Using a Hitachi 917 (Roche Diagnostics, Mannheim, Germany) reaction rate analyzer, levels of BAL total protein and activities of lactate dehydrogenase (LDH), alkaline phosphatase (ALP), γ-glutamyltransferase (GGT) and N-acetyl-β-glucosaminidase (NAG) were measured. Inflammatory cytokines (MCP-1, IL-8/CINC-1, M-CSF, osteopontin) in BAL were measured using ELISA test kits as described previously [50].

### Tissue analysis of barium content

Ba levels were measured in the lungs and lung-associated lymph nodes of exposed animals and controls. The lavaged lungs and aliquots of BAL of five animals per group were used. Barium content in the 4 week-exposure group lungs was measured three times (1, 2 and 35 days after the end of exposure) but only once (1 day post-exposure) in the 13-week exposure group. Each tissue sample was dried and sulfuric acid was added. The sample was then ashed and acid was vaporized at 500°C for 15 min. Sulfuric and nitric acid were added to the residue. Then a mixture of nitric acid, sulfuric acid and perchloric acid ( 2:1:1 v/v/v) was added and the solution was heated to oxidize organic matter. After evaporation, the residue was dissolved in concentrated sulfuric acid. The resulting solution was analyzed for ^137^Ba content by inductively coupled plasma mass spectrometry (ICP-MS) using Agilent 7500C (Agilent, Frankfurt, Germany). The limit of detection for Ba is 0.3 μg per tissue sample.

### Necropsy and histopathology

After 4 weeks of exposure, necropsy and histopathology were performed on selected rats at 1 day and 34 days after the end of exposure. Gross and histopathological examination of the lungs and extrapulmonary organs were performed on ten rats per group. The animals were euthanized by cutting the abdominal aorta and vena cava under sodium pentobarbital anesthesia. According to OECD no. 412, the following organs were weighed: adrenal glands, brain, heart, ovaries, uterus with cervix, kidney, liver, lungs, spleen, thymus, thyroid glands. The lungs were IT-instilled with neutral buffered 10% formalin at 30 cm water pressure. All other organs were fixed in the same fixative. The organs and tissues were trimmed, paraffin embedded and sectioned according to RITA trimming guides for inhalation studies [51-53]. Paraffin sections were stained with hematoxylin and eosin. Extrapulmonary organs and the respiratory tract, comprised of the nasal cavity (four levels), larynx (three levels), trachea (transverse and longitudinal with carina), lungs (five lobes), and mediastinal and tracheobronchial lymph nodes, were examined by light microscopy.

### Statistical analyses

#### Pharmacokinetic and single instillation studies

All BAL parameters and tissue ^131^Ba distribution data were analyzed using multivariate analysis of variance (MANOVA) followed by Bonferroni (Dunn) *post hoc* tests using SAS Statistical Analysis software (SAS Institute, Cary, NC). Lung clearance data were analyzed by linear regression of the natural logarithm of the lung ^131^BaSO_4_ levels (% dose) over time using R Program v. 3.1.0 (The R Foundation for Statistical Computing, Vienna, Austria).

#### Inhalation studies

Body weight differences were compared between BaSO_4_-exposed and control groups using Dunnett’s test. Bronchoalveolar lavage cytology, enzyme and cell mediator data were analyzed by non-parametric one-way analysis of variance using the Kruskal-Wallis test (two-sided). If the resulting p value was equal or less than 0.05, a pair-wise comparison of each test group with the control group was performed using the Wilcoxon test or the Mann–Whitney U-test. Comparison of organ weights was performed by nonparametric one-way analysis using the two-sided Kruskal–Wallis test, followed by a two-sided Wilcoxon test for the hypothesis of equal medians.

## Additional file

Additional file 1:
**Online Supporting Information.** **Table S1.** Physicochemical characterization of BaSO_4_ nanoparticles. **Table S2.** Distribution of recovered ^131^Ba post-intratracheal instillation of ^131^BaSO_4_ nanoparticles. **Table S3.** Distribution of recovered ^131^Ba post-gavage of ^131^BaSO_4_ nanoparticles. **Table S4.** Distribution of recovered ^131^Ba post-intravenous injection of ^131^BaSO_4_ nanoparticles. **Figure S1.** Structure of BaSO_4_ NM-220. A) Pore size distribution by Hg intrusion. B) Crystallinity by XRD (black line), with assignment of peaks to reference spectrum of bulk BaSO_4_ orthorombic (red). There were no unexpected peaks. **Figure S2.** Photocatalytic reactivity of A) NM-220 batch, B) reproduced batch: shown are UV–vis absorption spectra at 0, 2, 6 and 22 h incubation of Methylene Blue with BaSO_4_, irradiated with 1 mW/cm^2^ UV (350 nm) as specified in DIN 52980:2008–10, adapted for dispersed surfaces [1]. The blue curves are spectra of samples kept in the dark, the red-yellow curves are spectra of irradiated samples. The evaluation is compatible with zero degradation of the dye. **Figure S3.** Total lavaged neutrophils at 1 day post-instillation (A) or after the end of 4 and 13 weeks inhalation exposure of BaSO_4_ NPs (B). The x-axis represents the lung burden of barium at the end of 4-week (0.8 mg) or 13-week inhalation exposure (1.7 mg) (B). The neutrophil counts were significantly higher in instilled (A) than aerosol-exposed rats (B). **Figure S4.** Microscopic appearance of lungs after 4 weeks of inhalation exposure. Lung section of control animal (A) and animal exposed to 50 mg/m^3^ BaSO_4_ (B). **Figure S5.** Cumulative fecal and urinary excretion of ^131^Ba post-IV injection. Elimination of ^131^Ba was 17% via the feces (A) and only 4.4% of dose via the urine (B).

## Abbreviations

ALP: Alkaline phosphatase
BAL: Bronchoalveolar lavage
BALF: Bronchoalveolar lavage fluid
CINC: Cytokine-induced neutrophil chemoattractant
DLS: Dynamic light scattering
FaSSIF: Fasted state simulated intestinal fluid
GGT: γ-Glutamyl-transpeptidase
GSD: Geometric standard deviation
ICP-MS: Inductively coupled plasma mass spectrometry
IL: Interleukin
LDH: Lactate dehydrogenase
M-CSF: Macrophage colony stimulating factor
MCP: Monocyte chemoattractant protein
MMAD: Mass median aerodynamic diameter
MPO: Myeloperoxidase
NAG: N-Acetyl-β-glucosaminidase
NM: Nanomaterial
OPN: Osteopontin
PBS: Phosphate buffered saline
PMN: Polymorphonuclear
PSF: Phagolysosomal simulant fluid
PSLT: Poorly soluble low toxicity
SEM: Scanning electron microscopy
SMPS: Scanning mobility particle sizer
TEM: Transmission electron microscopy
TGA: Thermogravimetric analysis
