# Supplementary material for: Biokinetics and effects of barium sulfate nanoparticles
Source: Part Fibre Toxicol. 2014 Oct 21;11:55. doi: 10.1186/s12989-014-0055-3 (PMC4219084; doi:10.1186/s12989-014-0055-3)
Supplement: Additional file 1: — Online Supporting Information. Table S1. Physicochemical characterization of BaSO4 nanoparticles. Table S2. Distribution of recovered 131Ba post-intratracheal instillation of 131BaSO4 nanoparticles. Table S3. Distribution of recovered 131Ba post-gavage of 131BaSO4 nanoparticles. Table S4. Distribution of recovered 131Ba post-intravenous injection of 131BaSO4 nanoparticles. Figure S1. Structure of BaSO4 NM-220. A) Pore size distribution by Hg intrusion. B) Crystallinity by XRD (black line), with assignment of peaks to reference spectrum of bulk BaSO4 orthorombic (red). There were no unexpected peaks. Figure S2. Photocatalytic reactivity of A) NM-220 batch, B) reproduced batch: shown are UV–vis absorption spectra at 0, 2, 6 and 22 h incubation of Methylene Blue with BaSO4, irradiated with 1 mW/cm2 UV (350 nm) as specified in DIN 52980:2008–10, adapted for dispersed surfaces [1]. The blue curves are spectra of samples kept in the dark, the red-yellow curves are spectra of irradiated samples. The evaluation is compatible with zero degradation of the dye. Figure S3. Total lavaged neutrophils at 1 day post-instillation (A) or after the end of 4 and 13 weeks inhalation exposure of BaSO4 NPs (B). The x-axis represents the lung burden of barium at the end of 4-week (0.8 mg) or 13-week inhalation exposure (1.7 mg) (B). The neutrophil counts were significantly higher in instilled (A) than aerosol-exposed rats (B). Figure S4. Microscopic appearance of lungs after 4 weeks of inhalation exposure. Lung section of control animal (A) and animal exposed to 50 mg/m3 BaSO4 (B). Figure S5. Cumulative fecal and urinary excretion of 131Ba post-IV injection. Elimination of 131Ba was 17% via the feces (A) and only 4.4% of dose via the urine (B). [file 12989_2014_55_MOESM1_ESM.docx]

**Biokinetics and Effects of Barium Sulfate Nanoparticles**

**Online Supporting Information**

Nagarjun Konduru* ^a^, Jana Keller* ^b^, Lan Ma-Hock ^b^, Sibylle Gröters ^b^, Robert Landsiedel^§^ ^b^, Thomas C. Donaghey ^a^, Joseph D. Brain ^a^, Wendel Wohlleben ^b^, Ramon M. Molina^§^ ^a^

^a^Molecular and Integrative Physiological Sciences Program, Department of Environmental Health, Harvard School of Public Health, 665 Huntington Avenue, Boston, MA 02115, USA. ^b^BASF SE, Carl-Bosch-Straße 38, 67056 Ludwigshafen, Germany

* These authors contributed equally.

Corresponding Authors:

^§^ Ramon M. Molina, Harvard School of Public Health, Department of Environmental Health, Molecular and Integrative Physiological Sciences Program, 665 Huntington Avenue, Boston, MA 02115, USA. Tel: 1-617-432-2311, Email: rmolina@hsph.harvard.edu

^§^ Robert Landsiedel, Experimental Toxicology and Ecology, BASF SE, GV/TB - Z470, 67056 Ludwigshafen, Germany. Tel: +49 621 6056203, Email: robert.landsiedel@basf.com

| Table S1. Physicochemical characterization of BaSO_4_ nanoparticles | | |
| --- | --- | --- |
| Endpoints | BaSO_4_  NM-220 batch | BaSO_4_  reproduced batch |
| Particle size Distribution  TEM: primary particle diameter  State of Agglomeration  SEM: agglomerate diameter | 25 ± 10 nm  2,800 nm to 15,000 nm  sphere (SEM) | 25 ± 10 nm  2,000 nm to 13,000 nm  spheres (SEM) |
| Crystallite size (XRD) | 36.0 nm | 22.5 nm |
| Crystalline phase (XRD) | Barite orthorhombic | Barite orthorhombic |
| Specific surface area  Hg-intrusion porosimetry  DIN 66133 | surface area: 33 m²/g  pore sizes:  30 nm, 5000 nm | surface area: 38 m²/g  pore sizes:  30 nm, 200 nm, 5000 nm |
| Surface chemistry  XPS | Ba 13  O 52  C 17  S 11  Cl 3  P 3  N 1 | Ba 15  O 64  C 2  S 17  Na 2 |
| Surface charge  Electrophoretic mobility with  pH titration | IEP at pH 3.3 ± 0.2  ζ-pot at pH 7: -2.2(µm/s)/V/cm)  ZP: -28 ± 2 mV | IEP at pH 3.5 ± 0.2  ζ-pot at pH 7: -2.4(µm/s)/V/cm)  ZP: -32 ± 2 mV |
| Photocatalytic activity  DIN methylene blue assay | 1.1 + 0.2 -1.1 x 10^-3^  (compatible with zero) | 0.95 + 0.2 -0.95 x 10^-3^  (compatible with zero) |
| Dispersability  AUC, ISO13318: D50/AAN* | 116 nm/4.6 (in water)  285 nm/11 (in DMEM/FCS) | 160 nm/6.4 (in water)  198 nm/5.4 (in DMEM/FCS) |
| Solubility (ICP-MS)  in water 1 d  in DMEM/FCS 1d  in PSF 28 d  in PBS 28 d  in FaSSIF 7 d  in 0.1N HCl 1 d | See Fig. 1 for SEM results  0.05 wt%  0.02 wt%  0.1 wt% (recrystallizes)  0.1 wt%  0.1 wt%  1.0 wt% |  |
| Purity  (combined assessment from XRD, ICP-MS, XPS, TGA data) | 93.8 % | 95 % |
| Impurities | Water and organic  additives | Water and 1.8% organic additives (mass loss in TGA) |
| XRD, x-ray diffraction; XPS, x-ray photoelectron spectroscopy; AUC, analytical ultracentrifugation; ICP-MS, inductively coupled plasma mass spectrometry; DMEM/FCS,Dulbecco’s modified Eagle medium/fetal calf serum; PSF,phagolysosomal fluid; PBS, phosphate buffered saline; FaSSIF, fasted state simulant intestinal fluid; TGA, thermogravimetry; AAN, average agglomeration number  Data are mean ± standard deviation | | |

| Table S2. Distribution of recovered ^131^Ba post-intratracheal instillation of ^131^BaSO_4_ nanoparticles | | | | | | | | | | | | | | | | | | | | |
| --- | --- | --- | --- | --- | --- | --- | --- | --- | --- | --- | --- | --- | --- | --- | --- | --- | --- | --- | --- | --- |
|  | | 5 minutes | | | | 2 days | | | | 7 days | | | | 14 days | | | | 28 days | | |
| Blood | 2.52 | | ± | 1.73 | 0.078 | | ± | 0.02 | 0.04 | | ± | 0.01 | 0.00 | | ± | 0.00 | 0.07 | | ± | 0.07 |
| Plasma | 0.23 | | ± | 0.01 | 0.05 | | ± | 0.01 | 0.02 | | ± | 0.005 | 0.00 | | ± | 0.00 | 0.00 | | ± | 0.00 |
| RBC | 0.08 | | ± | 0.01 | 0.01 | | ± | 0.003 | 0.02 | | ± | 0.005 | 0.00 | | ± | 0.00 | 0.01 | | ± | 0.01 |
| TB LN | 0.03 | | ± | 0.01 | 0.05 | | ± | 0.02 | 0.09 | | ± | 0.04 | 0.03 | | ± | 0.02 | 0.01 | | ± | 0.01 |
| BM | 0.38 | | ± | 0.17 | 2.82 | | ± | 0.37 | 5.47 | | ± | 0.47 | 5.92 | | ± | 0.25 | 6.87 | | ± | 0.16 |
| Bone | 0.27 | | ± | 0.02 | 10.13 | | ± | 1.21 | 22.89 | | ± | 1.82 | 27.49 | | ± | 0.69 | 28.73 | | ± | 0.96 |
| Skin | 0.40 | | ± | 0.12 | 0.17 | | ± | 0.03 | 0.08 | | ± | 0.03 | 0.00 | | ± | 0.00 | 0.00 | | ± | 0.00 |
| Brain | 0.01 | | ± | 0.001 | 0.001 | | ± | 0.001 | 0.004 | | ± | 0.001 | 0.02 | | ± | 0.02 | 0.00 | | ± | 0.00 |
| SM | 1.12 | | ± | 0.19 | 0.01 | | ± | 0.003 | 0.01 | | ± | 0.002 | 0.001 | | ± | 0.001 | 0.00 | | ± | 0.00 |
| Testes | 0.01 | | ± | 0.001 | 0.004 | | ± | 0.001 | 0.001 | | ± | 0.001 | 0.00 | | ± | 0.00 | 0.00 | | ± | 0.00 |
| Kidneys | 0.05 | | ± | 0.01 | 0.01 | | ± | 0.003 | 0.01 | | ± | 0.001 | 0.00 | | ± | 0.00 | 0.00 | | ± | 0.00 |
| Spleen | 0.01 | | ± | 0.01 | 0.001 | | ± | 0.001 | 0.01 | | ± | 0.01 | 0.00 | | ± | 0.00 | 0.00 | | ± | 0.00 |
| Lungs | 97.2 | | ± | 1.47 | 79.67 | | ± | 2.02 | 52.82 | | ± | 5.40 | 27.35 | | ± | 1.38 | 16.14 | | ± | 1.11 |
| Heart | 0.01 | | ± | 0.001 | 0.002 | | ± | 0.001 | 0.002 | | ± | 0.001 | 0.00 | | ± | 0.00 | 0.00 | | ± | 0.00 |
| Liver | 0.04 | | ± | 0.01 | 0.01 | | ± | 0.003 | 0.01 | | ± | 0.003 | 0.00 | | ± | 0.00 | 0.00 | | ± | 0.00 |
| Stomach | 0.03 | | ± | 0.01 | 0.12 | | ± | 0.03 | 0.06 | | ± | 0.01 | 0.04 | | ± | 0.01 | 0.00 | | ± | 0.00 |
| SI | 0.12 | | ± | 0.02 | 0.52 | | ± | 0.04 | 0.23 | | ± | 0.04 | 0.11 | | ± | 0.01 | 0.01 | | ± | 0.01 |
| LI | 0.02 | | ± | 0.005 | 0.29 | | ± | 0.06 | 0.21 | | ± | 0.04 | 0.11 | | ± | 0.03 | 0.02 | | ± | 0.01 |
| Cecum | 0.01 | | ± | 0.004 | 0.55 | | ± | 0.06 | 0.44 | | ± | 0.05 | 0.14 | | ± | 0.02 | 0.04 | | ± | 0.01 |
| Total | 100 | | ± | 1.83 | 101.8 | | ± | 2.28 | 103.0 | | ± | 3.27 | 88.9 | | ± | 1.64 | 86.2 | | ± | 1.91 |
| Urine |  | |  |  | 0.69 | | ± | 0.07 | 2.63 | | ± | 0.27 | 3.09 | | ± | 0.11 | 3.95 | | ± | 0.04 |
| Feces |  | |  |  | 6.63 | | ± | 0.35 | 17.98 | | ± | 0.51 | 24.62 | | ± | 0.50 | 30.43 | | ± | 0.19 |
| Data are % of instilled dose (mean ± standard error of the mean, n = 5 rats per group)  RBC, red blood cells; TB LN, tracheobronchial lymph nodes; BM, bone marrow; SM, skeletal muscle; SI, small intestine; LI, large intestine; Total, sum of all analyzed organs. | | | | | | | | | | | | | | | | | | | | |

| Table S3. Distribution of recovered ^131^Ba post-gavage of ^131^BaSO_4_ nanoparticles | | | | | | |
| --- | --- | --- | --- | --- | --- | --- |
|  | 5 minutes | | | 7 days | | |
| Blood | 0.01 | ± | 0.01 | 0.05 | ± | 0.025 |
| Plasma | 0.002 | ± | 0.0004 | 0.004 | ± | 0.002 |
| RBC | 0.002 | ± | 0.001 | 0.002 | ± | 0.002 |
| TB LN | 0.14 | ± | 0.13 | 0.00 | ± | 0.00 |
| BM | 0.01 | ± | 0.005 | 0.07 | ± | 0.06 |
| Bone | 0.01 | ± | 0.005 | 0.08 | ± | 0.01 |
| Skin | 0.01 | ± | 0.005 | 0.002 | ± | 0.001 |
| Brain | 0.001 | ± | 0.0002 | 0.00 | ± | 0.00 |
| SM | 0.01 | ± | 0.01 | 0.00 | ± | 0.00 |
| Testes | 0.0005 | ± | 0.0002 | 0.00 | ± | 0.00 |
| Kidneys | 0.001 | ± | 0.001 | 0.00 | ± | 0.00 |
| Spleen | 0.003 | ± | 0.002 | 0.00 | ± | 0.00 |
| Lungs | 0.11 | ± | 0.08 | 0.00 | ± | 0.00 |
| Heart | 0.003 | ± | 0.003 | 0.00 | ± | 0.00 |
| Liver | 0.004 | ± | 0.001 | 0.00 | ± | 0.00 |
| Stomach | 99.66 | ± | 3.38 | 0.004 | ± | 0.004 |
| SI | 0.02 | ± | 0.01 | 0.001 | ± | 0.0004 |
| LI | 0.01 | ± | 0.004 | 0.001 | ± | 0.001 |
| Cecum | 0.01 | ± | 0.003 | 0.003 | ± | 0.001 |
| Total | 99.999 | ± | 3.26 | 99.22 | ± | 1.39 |
| Urine |  |  |  | 0.02 | ± | 0.002 |
| Feces |  |  |  | 99.03 | ± | 1.38 |
| Data are % of gavaged dose (mean ± standard error of the mean, n = 5 rats per group)  RBC, red blood cells; TB LN, tracheobronchial lymph nodes; BM, bone marrow; SM, skeletal muscle; SI, small intestine; LI, large intestine; Total, sum of all analyzed organs. | | | | | | |

| Table S4. Distribution of recovered ^131^Ba post-intravenous injection of ^131^BaSO_4_ nanoparticles | | | | | | | | | |
| --- | --- | --- | --- | --- | --- | --- | --- | --- | --- |
|  | 2 hours | | | 2 days | | | 7 days | | |
| Blood | 0.36 | ± | 0.04 | 0.02 | ± | 0.01 | 0.04 | ± | 0.04 |
| Plasma | 0.20 | ± | 0.01 | 0.04 | ± | 0.01 | 0.001 | ± | 0.001 |
| RBC | 0.09 | ± | 0.01 | 0.04 | ± | 0.02 | 0.00 | ± | 0.00 |
| TB LN | 0.03 | ± | 0.003 | 0.03 | ± | 0.01 | 0.07 | ± | 0.02 |
| BM | 1.30 | ± | 0.05 | 6.24 | ± | 0.48 | 10.71 | ± | 0.50 |
| Bone | 3.93 | ± | 0.16 | 24.08 | ± | 1.34 | 46.34 | ± | 2.31 |
| Skin | 0.72 | ± | 0.05 | 0.38 | ± | 0.06 | 0.10 | ± | 0.03 |
| Brain | 0.02 | ± | 0.004 | 0.02 | ± | 0.01 | 0.02 | ± | 0.01 |
| SM | 0.05 | ± | 0.004 | 0.06 | ± | 0.01 | 0.05 | ± | 0.01 |
| Testes | 0.02 | ± | 0.001 | 0.005 | ± | 0.01 | 0.01 | ± | 0.01 |
| Kidneys | 0.33 | ± | 0.01 | 0.35 | ± | 0.03 | 0.20 | ± | 0.02 |
| Spleen | 3.01 | ± | 0.57 | 2.79 | ± | 0.32 | 1.27 | ± | 0.14 |
| Lungs | 10.12 | ± | 0.92 | 17.50 | ± | 1.74 | 8.19 | ± | 0.74 |
| Heart | 0.06 | ± | 0.01 | 0.05 | ± | 0.01 | 0.04 | ± | 0.01 |
| Liver | 57.97 | ± | 1.11 | 46.15 | ± | 2.09 | 11.73 | ± | 1.16 |
| Stomach | 0.12 | ± | 0.01 | 0.11 | ± | 0.004 | 0.09 | ± | 0.01 |
| SI | 0.78 | ± | 0.06 | 0.51 | ± | 0.02 | 0.18 | ± | 0.01 |
| LI | 0.04 | ± | 0.01 | 0.39 | ± | 0.10 | 0.19 | ± | 0.01 |
| Cecum | 0.05 | ± | 0.005 | 0.59 | ± | 0.07 | 0.28 | ± | 0.02 |
| Total | 78.85 | ± | 1.82 | 108.55 | ± | 1.93 | 100.79 | ± | 2.62 |
| Urine |  |  |  | 2.77 | ± | 0.12 | 4.36 | ± | 0.28 |
| Feces |  |  |  | 6.46 | ± | 0.13 | 16.96 | ± | 0.20 |
| Data are % of injected dose (mean ± standard error of the mean, n = 5 rats per group)  RBC, red blood cells; TB LN, tracheobronchial lymph nodes; BM, bone marrow; SM, skeletal muscle; SI, small intestine; LI, large intestine; Total, sum of all analyzed organs. | | | | | | | | | |


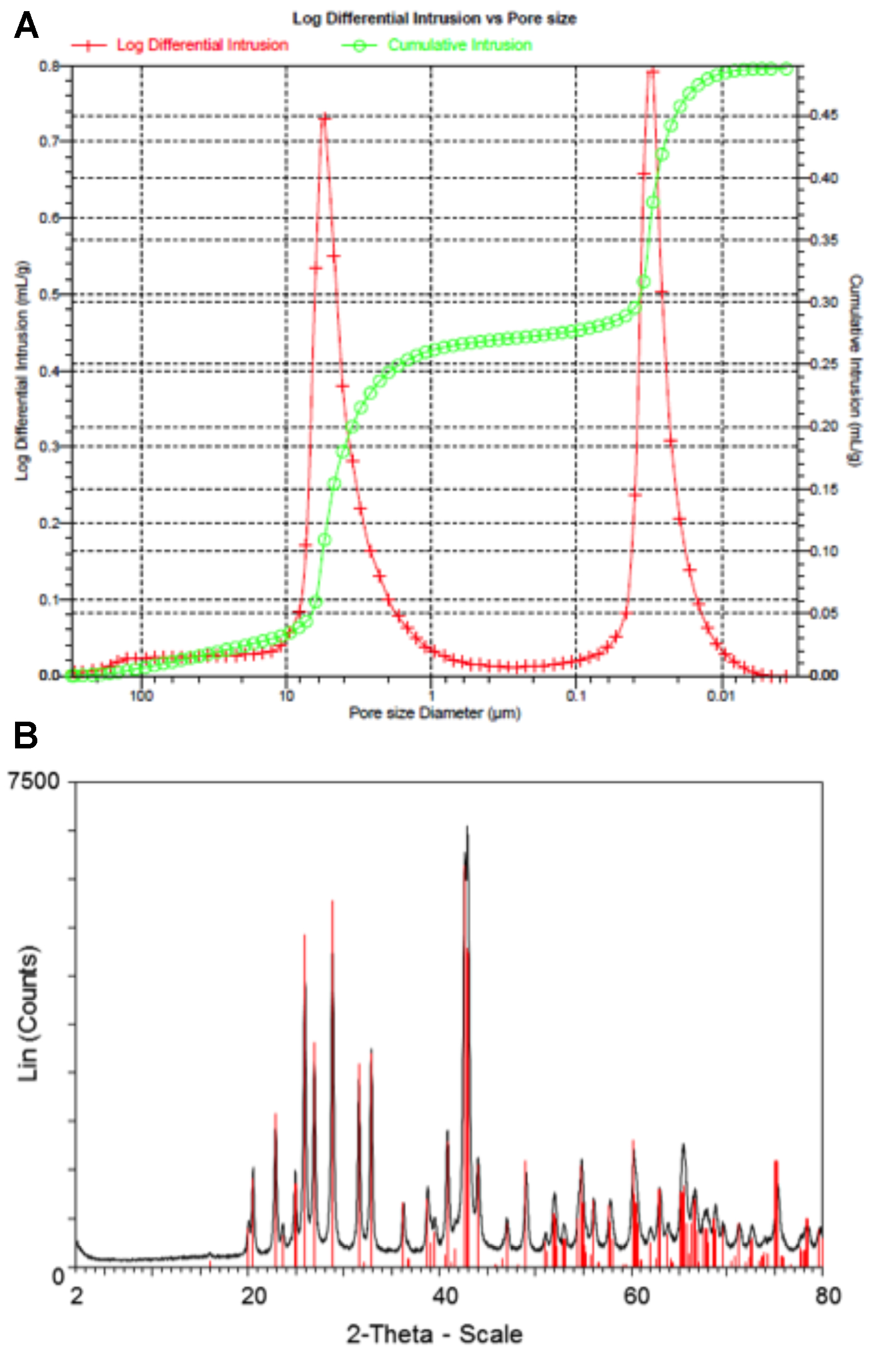


Figure S1. Structure of BaSO_4_ NM-220. A) Pore size distribution by Hg intrusion.
B) Crystallinity by XRD (black line), with assignment of peaks to reference spectrum of bulk BaSO_4_ orthorombic (red). There were no unexpected peaks.


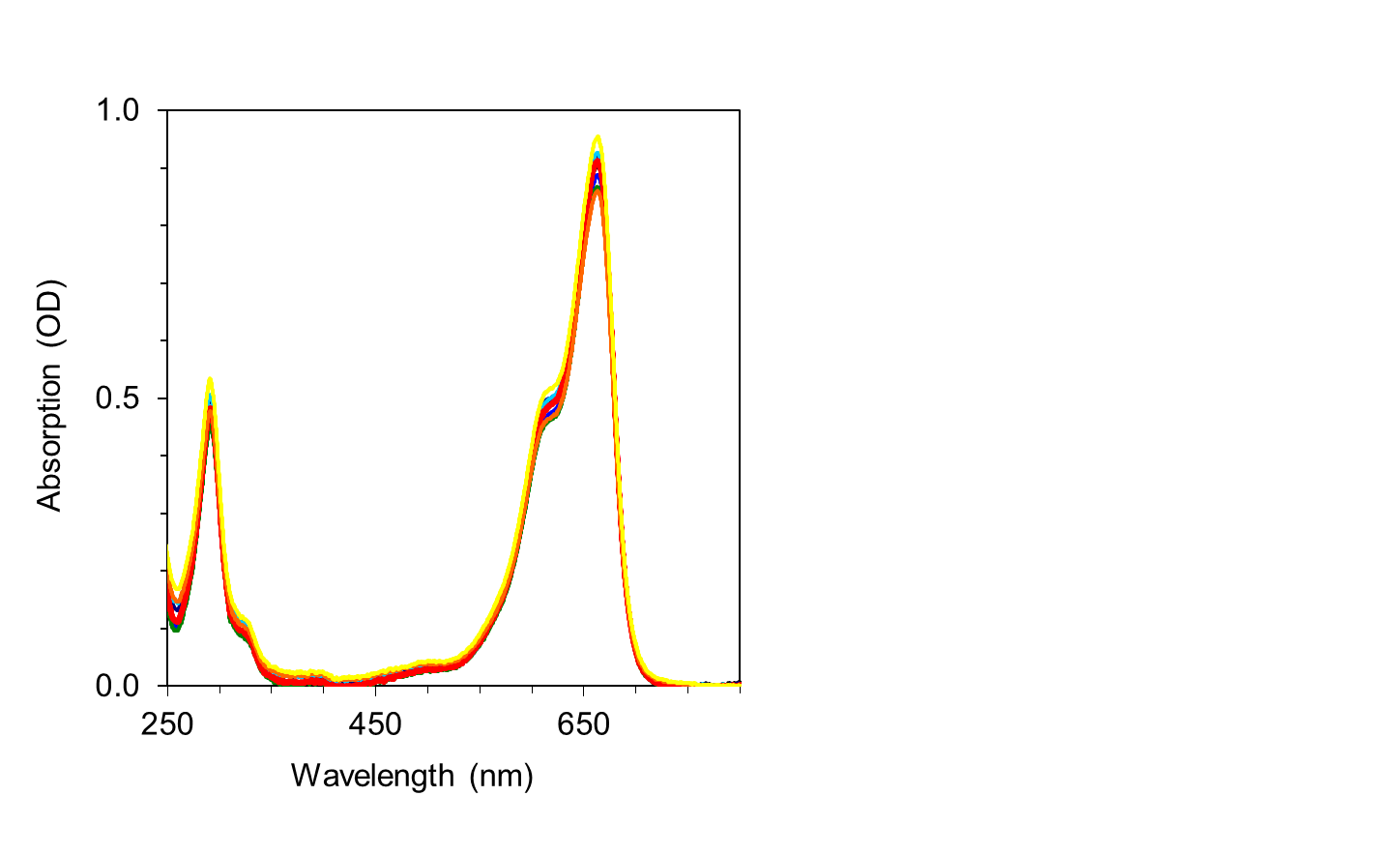


Figure S2. Photocatalytic reactivity of A) NM-220 batch, B) reproduced batch: shown are UV-Vis absorption spectra at 0, 2, 6 and 22 h incubation of Methylene Blue with BaSO_4_, irradiated with 1 mW/cm² UV (350 nm) as specified in DIN 52980:2008-10, adapted for dispersed surfaces [[1](#_ENREF_1)]. The blue curves are spectra of samples kept in the dark, the red-yellow curves are spectra of irradiated samples. The evaluation is compatible with zero degradation of the dye.

Figure S3. Total lavaged neutrophils at 1 day post-instillation (A) or after the end of 4 and 13 weeks inhalation exposure of BaSO_4_ NPs. The x-axis represents the lung burden of barium at the end of 4-week (0.8 mg) or 13-week inhalation exposure (1.7 mg) (B). The neutrophil counts were significantly higher in instilled (A) than aerosol-exposed rats.


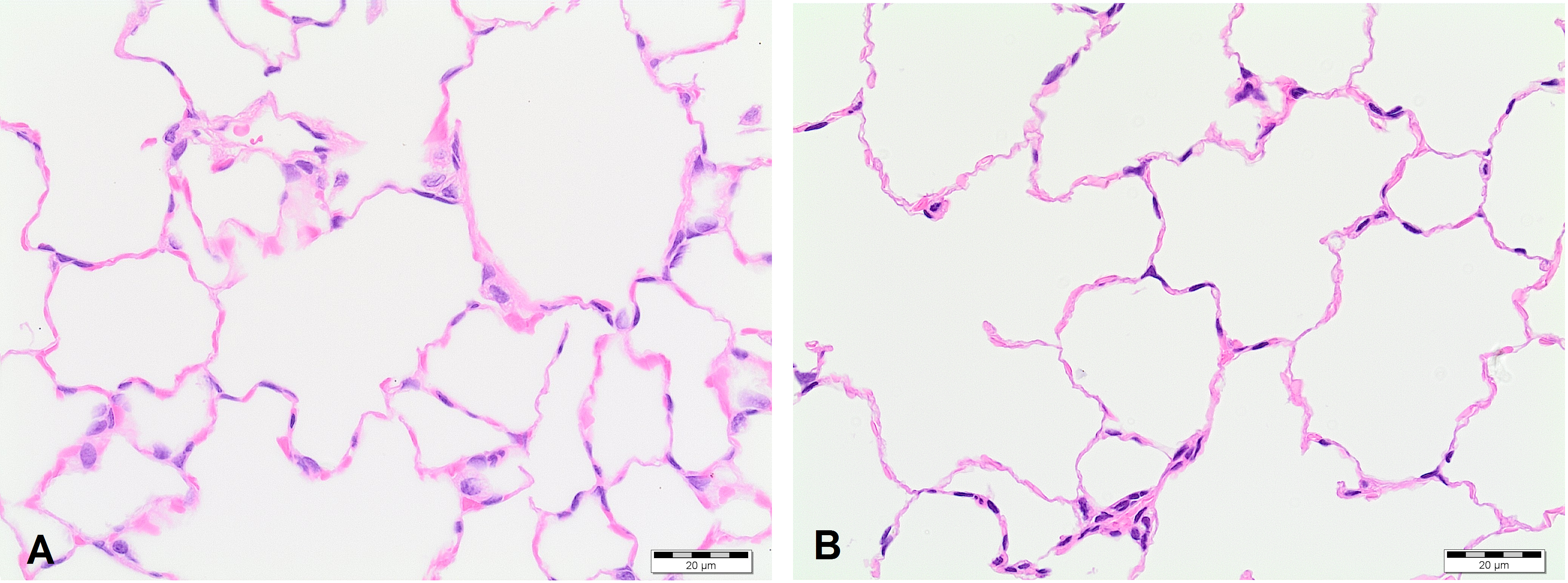


Figure S4. Microscopic appearance of lungs after 4 weeks of inhalation exposure. Lung section of control animal (A) and animal exposed to 50 mg/m³ BaSO_4_ (B).

Figure S5. Cumulative fecal and urinary excretion of ^131^Ba post-IV injection. Elimination of ^131^Ba was 17% via the feces (A) and only 4.4% of dose via the urine (B).

**Reference**

1 Wohlleben, W., Ma-Hock, L., Boyko, V., Cox, G., Egenolf, H., Freiberger, H., Hinrichsen, B., Hirth, S., Landsiedel, R.: **Nanospecific guidance in REACH: A comparative physical-chemical characterization of 15 materials with methodical correlations.** *J.Ceram.Sci.Tech* 2013, **4**: 93-104.
